# A murine specific expansion of the *Rhox *cluster involved in embryonic stem cell biology is under natural selection

**DOI:** 10.1186/1471-2164-7-212

**Published:** 2006-08-17

**Authors:** Melany Jackson, Alistair J Watt, Philippe Gautier, Derek Gilchrist, Johanna Driehaus, Gerard J Graham, Jon Keebler, Franck Prugnolle, Philip Awadalla, Lesley M Forrester

**Affiliations:** 1 John Hughes Bennett Laboratory, University of Edinburgh, Western General Hospital, Edinburgh, Scotland, EH4 2XU, UK; 2 Division of Immunology, Infection and Inflammation, University of Glasgow, Glasgow, Scotland, G12 8QQ, UK; 3 MRC Human Genetics Unit, Western General Hospital, Edinburgh, Scotland, EH4 2XU, UK; 4 Department of Genetics, North Carolina State University, Raleigh, NC, USA

## Abstract

**Background:**

The rodent specific reproductive homeobox (*Rhox*) gene cluster on the X chromosome has been reported to contain twelve homeobox-containing genes, *Rhox1-12*.

**Results:**

We have identified a 40 kb genomic region within the *Rhox *cluster that is duplicated eight times in tandem resulting in the presence of eight paralogues of *Rhox2 *and *Rhox3 *and seven paralogues of *Rhox4*. Transcripts have been identified for the majority of these paralogues and all but three are predicted to produce full-length proteins with functional potential. We predict that there are a total of thirty-two *Rhox *genes at this genomic location, making it the most gene-rich homoeobox cluster identified in any species. From the 95% sequence similarity between the eight duplicated genomic regions and the synonymous substitution rate of the *Rhox2*, *3 *and *4 *paralogues we predict that the duplications occurred after divergence of mouse and rat and represent the youngest homoeobox cluster identified to date. Molecular evolutionary analysis reveals that this cluster is an actively evolving region with *Rhox2 *and *4 *paralogues under diversifying selection and *Rhox3 *evolving neutrally. The biological importance of this duplication is emphasised by the identification of an important role for *Rhox2 *and *Rhox4 *in regulating the initial stages of embryonic stem (ES) cell differentiation.

**Conclusion:**

The gene rich *Rhox *cluster provides the mouse with significant biological novelty that we predict could provide a substrate for speciation. Moreover, this unique cluster may explain species differences in ES cell derivation and maintenance between mouse, rat and human.

## Background

Homeobox genes encode transcription factors defined by a 60 amino acid homeodomain motif and have fundamental roles in many aspects of biology [1-4]. The most studied example of these are the *Hox *genes which have an ancestral role in the patterning of the primary body axis and in vertebrates have adopted additional roles in a number of processes including limb and genital development [5-7]. In the majority of species, *Hox *genes are further defined by their clustered organisation in the genome. For example, in *Drosophila*, eight *Hox *genes are present in a single cluster whereas in mammals, four clusters exist of up to twelve genes on four separate chromosomes [8]. The clustered organisation of *Hox *genes is crucial to their function. *Hox *genes show colinearity of expression where the relative position of the *Hox *genes along the cluster correlates with the time and domain of gene expression along the anterior -posterior axis of the body [9]. The maintenance of *Hox *clusters has provided a model of evolution by gene duplication, an essential source of material for the generation of novel gene function. It is predicted that, initially, newly duplicated genes are functionally redundant. Three different evolutionary outcomes exist which will resolve this redundancy. Duplicate genes can either be lost by degenerative mutations (nonfunctionalization), functionally compromised in a complementary fashion such that the duplicated genes are functionally equivalent to the single copy ancestral gene (subfunctionalisation) or acquire novel function through natural selection of beneficial mutations (neofunctionalization). *Hox *clusters provide evidence for all three evolutionary processes [10,11].

Recently, a novel homeobox gene cluster (*Rhox*) was discovered on the X chromosome containing 12 genes (*Rhox1*-*12*). *Rhox *genes are primarily expressed in reproductive tissues and placenta with additional expression domains in endodermal derived tissues [3]. *Rhox5 *is essential for the production and motility of sperm [3] and we have shown that *Rhox4 *plays an important role in the early stages of ES cell differentiation [12]. It was reported that the *Rhox *cluster also displays colinearity with the level and timing of expression during spermatogenesis of subsets of *Rhox *genes consistent with their position within specific sub-clusters [3]. Interestingly, the *Rhox *cluster appears to be rodent specific with only two *Rhox *homologues identified in humans leading to speculation that the cluster is involved in the increased reproductive capacity of rodents compared to humans [3].

We describe an extensive duplication within the murine *Rhox *cluster consisting of eight tandem repeats of a 40 kilobase (kb) unit containing *Rhox2*, *3 *and *4 *potentially increasing the number of *Rhox *genes in this cluster to thirty-two. Transcripts have been identified for the majority of these paralogues and all but three are predicted to produce full-length proteins. Sequence and evolutionary analyses reveal significant differences in the evolutionary signatures of *Rhox 2,3 *and *4 *paralogues indicative of distinct selection pressures. We have performed functional studies in ES cells that strongly support a role for both *Rhox2 *and *Rhox4 *in embryonic stem cell biology.

## Results

### Genomic structure of the duplicated *Rhox *α sub-cluster

**Figure 1:**
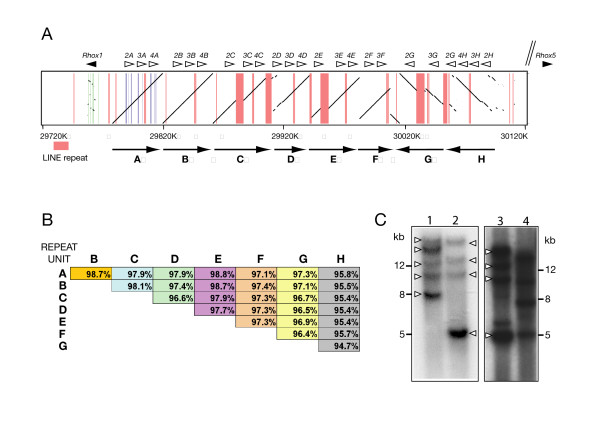
A. Dotplot analysis comparing repeat A to repeats B to H using the NCBI m33 mouse assembly (freeze May 27, 2004, strain C57BL/6J). Black lines represent regions of identity interspersed with LINEs (horizontal pink lines). The orientation and approximate size of each repeat unit is presented as black arrows. The relative position and orientation of *Rhox1 *(green lines, black arrowhead) and *Rhox2*, *3 *and *4 *(blue lines, white arrowhead) and Rhox5 (which is upstream of this region, black arrowhead) are shown. The X chromosome co-ordinates are provided below the dotplot. B. BLAST analysis comparing genomic sequence of each repeat to all others. LINE repeats and rearrangements were not added. Top row represents a comparison of repeat A to B-H, second row repeat B to C-H and so on. C. Southern Blot analysis. CGR8 ES cell genomic DNA digested with Asp718 (lane 1) and HindIII (lane2) and hybridised to *Rhox4 *exon 1 and intron 1 probe. Multiple bands can be seen with both digests (arrowed) of approximately 20, 13, 10 and 5 kb (HindIII) and 23, 18, 13, 10, and 8 kb (Asp718). *Mus domesticus *(lane 3) and *Mus spretus *(lane 4) genomic DNA digested with HindIII and hybridised to a *Rhox4 *exon 2 probe. Fragment sizes common to the *Mus musculus *(ES cells) and *Mus domesticus *are arrowed. Additional bands are predicted to be due to the differences between the probes used. Positions of size standards are provided in kilobases.

In the course of a detailed analysis of the *Rhox4 *gene from the mouse genome assembly, we identified multiple copies of *Rhox2*, *Rhox3 *and *Rhox4 *spanning approximately 350 kb of the X chromosome at region A2 from position 29780 K to 30100 K. Dotplot analysis of this genomic region identified a tandem segmental duplication composed of an approximately 40 kb unit repeated eight times; six in the forward orientation (A-F) and two in reverse orientation (G&H) (Figure 1A). Each of these duplications contain a single copy of *Rhox2, 3 and 4 *with the exception of repeat F which lacks *Rhox4 *due to a truncation of the 3 prime end. There are therefore eight paralogues of *Rhox2 *and *Rhox3 *and seven of *Rhox4 *(Figure 1A) producing 32 *Rhox *genes in total. Variation exists between each repeat that is primarily due to small rearrangements and repeat element insertions, particularly long interspersed nuclear elements (LINEs). Outwith these features, BLAST analysis reveals greater than 95% similarity between each repeat, the majority of which is intergenic sequence (Figure 1B). From the level of similarity and the small rearrangements and repeat element insertions between the different units, one can make the hypothesis that there were several rounds of duplication with the oldest repeats being at the 3 prime end. Indeed for the two reverse repeat units at the 3 prime region it is difficult to define their boundaries accurately because of more extensive rearrangements. The organisation of this duplication is consistent with other rodent segmental duplications that are largely tandem or tightly clustered [13]. The maintenance of orientation and spacing between the different paralogues of *Rhox2*, *3*, and *4 *suggests that it is unlikely that any of the paralogues have arisen independently of the 40 kb tandem segmental duplications.

Given such high similarity between the duplications, we sought to eliminate the possibility of improperly assembled database sequence using Southern Blot analysis. Genomic DNA from CGR8 ES cells was digested with HindIII and Asp718, two enzymes that are predicted to produce significantly different fragment sizes when probed with *Rhox4 *sequence spanning exon1 and intron1. The *Rhox4 *probe hybridises to multiple HindIII fragments of approximately 20, 13, 10 and 5 kb with Asp718 producing 23, 18, 13, 10, and 8 kb fragments (Figure 1C) that are consistent with the predicted sizes from the database sequence. Identical results were observed for genomic DNA isolated from the *Mus musculus *strains C57/Bl6, Balb/c and FVB. Southern blot analysis of genomic DNA from *Mus domesticus *and *Mus spretus *identified multiple fragments hybridising to *Rhox4 *consistent with multiple duplications of the *Rhox *cluster in these species. These data suggest that the database sequence represents genuine genomic duplications and are highly unlikely to be artefacts of the genomic sequence build.

### Predicted cDNA and protein sequences encoded by duplicated *Rhox *genes

**Figure 2:**
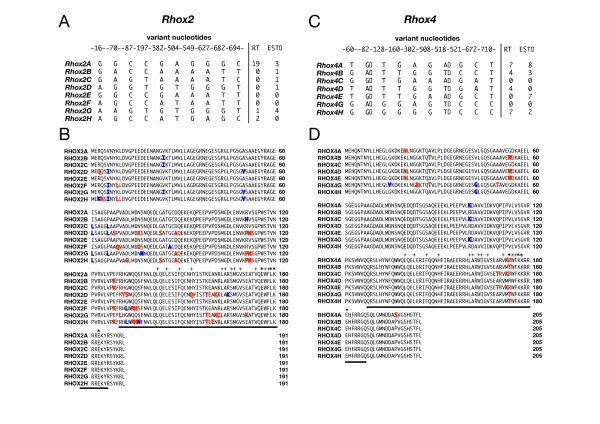
A & C. Unique nucleotide profile and relative expression of the predicted *Rhox2A-H *(A) or *Rhox4A-H *(C) transcripts. The position of the nucleotides is relative to the published start sites. Column RT and EST refers to the number of each transcript detected by RT-PCR or in the EST database respectively that matched this profile. B & D. ClustalW alignment of predicted amino acid sequence from *Rhox2A-H *(B) or *Rhox4A-H *(D) Residues different from the consensus are highlighted in blue (conservative amino acid change) or red (non-conservative amino acid change). The homeobox domain is underlined with hydrophobic residues (+) crucial for homeodomain packing and residues involved in DNA binding (*) annotated.

We analysed the gene structure and the predicted cDNA and amino acid sequence of each *Rhox *paralogue. The similarity between each of the duplications is manifest in the preservation of intron-exon structure between each variant of *Rhox2*, *3 *and *4 *[see Additional file 1]. Clustal W comparison of the cDNA sequences that were predicted from repeats A-H revealed, as with the overall duplication unit similarity, greater than 95% similarity between *Rhox2A-H*, *Rhox3A-H *and *Rhox4A-H *[see Additional file 2]. Despite such a high similarity we were able to build up a unique nucleotide profile of each copy of *Rhox2*, *3*and *4 *allowing them to be definitively identified (Figure 2A, C, [see Additional file 3]).

We focussed on *Rhox2A-H *and *Rhox4A-H *and used these nucleotide profiles to determine whether these paralogues were expressed. Using primer sequences common to all copies of *Rhox2 *or *Rhox4*, we cloned and sequenced RT-PCR products derived from RNA isolated from differentiating ES cells and placenta, cell types known to express *Rhox2 *and *4*. In addition, the mouse subset database of expressed sequence tags (est_mouse) was analysed for the unique nucleotide profile of each paralogue. Using both techniques, the expression of six out of eight paralogues of *Rhox2 *(A, B, C, D, G, H) and five out of seven paralogues of *Rhox4 *(A, B, D, E, H) were confirmed (Figure 2A, C). Translation of the predicted cDNAs for each of *Rhox2*, 3 and *4 *paralogue is presented in Figure 2B, D and [see Additional file 1]. ClustalW analysis of RHOX2, 3 and 4 paralogues identifies most variation in protein sequence within the RHOX2 paralogues with RHOX3 showing the least variation (Figure 2; [see Additional files 2 and 3]). Both RHOX2 and RHOX4 groups have non-conservative amino acid variations within the homeodomain region including residues crucial for homeodomain packing or DNA binding [9] (Figure 2B, D). Such protein sequence variation could provide functional variation between the RHOX2 and RHOX4 paralogues with, for example, changes in homeodomain sequence leading to different DNA binding characteristics. Alternatively, such changes may reflect the nonfunctionalisation of paralogues due to disrupted DNA binding. RHOX3B, D and G contain non-sense mutations that truncate the predicted proteins after 9 (B and G) or 174 (D) amino acids [see Additional file 3]. These three genes are unlikely to be functional due to the absence of the homeodomain indicating that these paralogues have become pseudogenes.

These data, therefore, predicts that eight of the *Rhox2*, five of the *Rhox3 *and seven of the *Rhox4 *paralogues are capable of producing full-length proteins. Therefore, of the thirty-two genes in the *Rhox *cluster, at least twenty-nine are predicted to be functional which is over twice the number of genes present in the largest homeobox cluster identified to date in any species.

### Molecular evolution of *Rhox2*, *3 *and *4*

*Rhox2*, *3 *and *4 *are in tandem alignment with each other and show some sequence similarity. However, nucleotide similarity is low; for example, *Rhox2 *and *Rhox4 *are approximately 65% similar and are, therefore, evolving as separate loci. Given that the paralogues present on each duplication unit arose simultaneously, this could be suggestive of different evolutionary pressures on the paralogues within each duplication unit. To test this hypothesis we calculated maximum likelihood estimates of rates of non-synonymous (*dN*) and synonymous substitution (*dS*) among alignments of *Rhox2*, *3 *and *4 *paralogues using a codon-based model of sequence evolution. These models allowed both the analysis of branch specific ratios of the rates of *dN *and *dS*, or codon specific ratios along the sequence, following the methods proposed by Yang and colleagues [14,15] respectively. Generally, it is assumed that synonymous substitutions among lineages (genes) accumulate through a neutral or nearly neutral process, and by comparing this rate of accumulation to that for non-synonymous substitutions it is possible to ask whether the non-synonymous substitutions accumulate more (diversifying or positive selection) or less (constrained) than the neutral rate. Effectively a *dN/dS *rate of one is neutral, *dN/dS *significantly greater than one suggests positive or diversifying selection and a significant *dN/dS *of less than one indicates amino-acid constraint. We calculated these ratios, henceforth called ω, for the whole gene for each lineage to ask whether particular lineages (duplicates) were evolving under positive selection and for each codon across all lineages in order to ask whether a codon was under positive selection.

The estimates of *dN*, *dS *and ω across each set of paralogues of *Rhox2*, *3 *and *4 *are given in Table 1. Both the *dN *and *dS *rates are relatively low particularly in comparison to genome-wide estimates of divergence between *Mus musculus *and *Rattus norvegicus *confirming that these duplications are relatively recent [16]. Moreover, there is a 10-fold difference in the synonymous substitution rates between *Rhox2 *and *Rhox3 *or *Rhox4*. Given that the gene paralogues were duplicated together as single genomic fragments this suggests that the rate of nucleotide change is significantly different between *Rhox2*, *3 *and *4*.

To confirm that the ω values are different between *Rhox *genes, we selected *Rhox2 *and *Rhox4 *and asked whether they are evolving differently after aligning the *Rhox2 *and *Rhox4 *paralogues to each other. Allowing the *Rhox2 *genes to have one ratio and the *Rhox4 *genes to have another ratio, we found that the likelihood (*l*) of a model with different ratios (*l *= -1838.04, number of parameters (np) = 31) significantly differed from a model where all the *Rhox *genes had the same ratio (*l *= -1844.17 np = 29, 2(Δ*l*) = 12.26 p-value < 0.01 with df = 2). Taken together, this data shows that the *Rhox *loci, as part of a relatively recently duplicated genomic region, are under different evolutionary selection.

**Figure 3:**
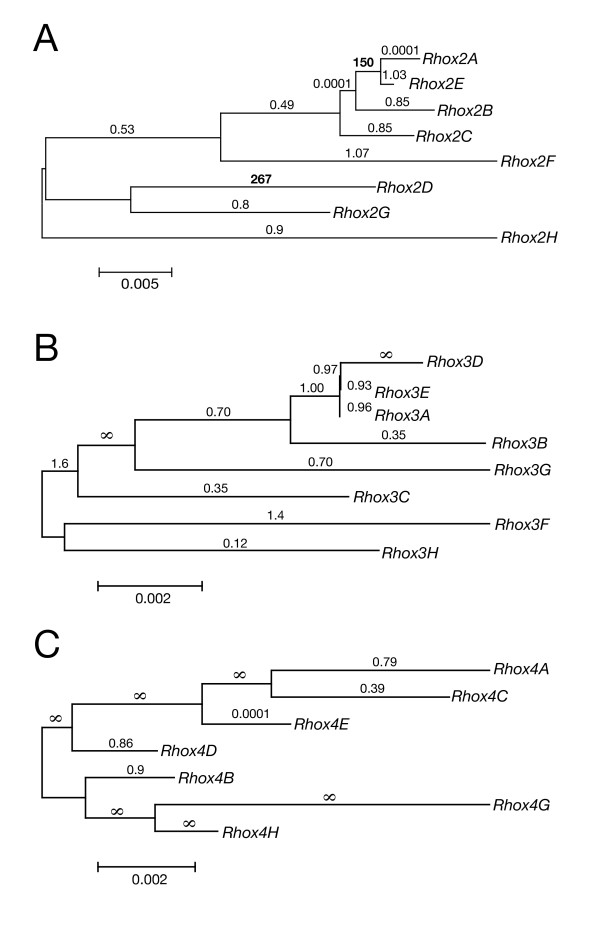
A, B & C. Phylogeny of the *Rhox2 *(A), *Rhox3 *(B) and *Rhox4 *(C) paralogues. The numbers shown along each branch are the maximum likelihood estimates of the ratio of nonsynonymous and synonymous substitutions for the entire gene along that branch. Data for a "free-ratio" model is shown which allows for a different dN/dS ratio (parameter ω) for each branch in the tree. Branches are drawn in proportion to estimates of their lengths.

Given that there was significant variation among the *Rhox *loci, it seemed reasonable to test whether ω values were the same among the individual paralogues within each of *Rhox2*, *3*, and *4*. Given a tree for each set of genes (Figure 3A) we estimated substitution rates along each lineage. For *Rhox2*, the one-ratio model, which assumes the same ω parameter for the entire tree, leads to *l*_0 _= -1242.17. We performed analysis of models allowing all branches to have different ω values and we also placed different constraints on the different branches, particularly for branches labelled A and B; (Figure 3A): ω_A_, ω_B_, ω_0_. Ratios for branches A and B (ω_A _= ω_B_) are significantly greater than the background ratio (2Δ *l *= 10.32 p < 0.05) and also significantly greater than one. However, a model allowing all branches to vary did not significantly differ from the one ratio model.

Among the *Rhox4 *paralogues (Figure 3C) mostly non-synonymous substitutions were observed. Again, the log-likelihood difference between the one-ratio model and the free-ratio model suggests that there are no significant differences between the ratios of different branches (2Δ *l *= 6.16, df = 10, 0.50 < p-value < 0.90). This analysis is limited as no ω values can be calculated for certain lineages due to the absence of synonymous substitutions. For *Rhox3 *paralogues B, D and G have in-frame stop codons and we would expect mutations to accumulate in a neutral fashion in these paralogues if they have lost function. The comparison of the free-ratio model and the one-ratio model indicates that there are no differences among branches (2Δ *l *= 5.33, df = 12, 0.50 < p-value < 0.90) (Figure 3B).

**Figure 4:**
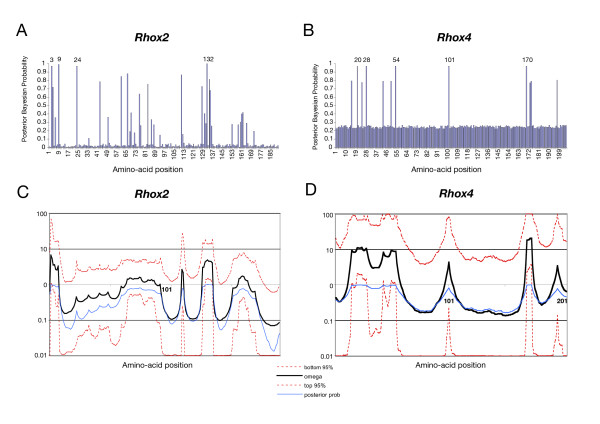
A & B. Posterior Probability of site classes along the *Rhox2 *(A) and *Rhox4 *(B) coding aligments. Model M2 is used. Only probabilities of w2 are shown. Sites with a posterior probability higher than 0.95 are considered as having dN/dS ratios significantly greater than 1. C & D. Variation in ω across the *Rhox2 *(C) and *Rhox4 *(D) genes was modelled using Omegamap 17. We averaged over the number and position of the blocks using reversible-jump MCMC to obtain the posterior distribution of the omega. The dotted lines are the top and bottom 95% of the estimates of ω from 500,000 iterations. The black line is ω per codon smoothed over blocks of undefined size. Posterior probabilities of >0.95 (blue line) likely indicate significant ω or positive selection. The location of the largest ω values coincide with similar locations as shown in Figure 4A & B obtained from PAML analyses (see text).

We next tested for variable ω among codons. The strict neutral model assumes that a proportion p_0 _of sites are conserved with ω _0 _= 0 and a proportion *p*_1 _= 1- *p*_0 _are neutral with ω_1 _= 1 fitting the data better than a strict one-ratio model which assumes the same ratio for all sites. The alternative model allows for variable ω among codons, with some codons having ω_1_> 1 (diversifying positive selection). We used a likelihood (PAML) and a bayesian approach [17] to test for a departure from neutrality among codons. The likelihood approach assumes no recombination (one phylogenetic history) whereas the bayesian approach allows for recombination to occur among lineages throughout the sequence (and hence, independent multiple genealogies). Using PAML, all models that allow for positively selected sites suggest existence of such sites among *Rhox2 *and *Rhox4 *paralogues (Figure 4A, B). For example, the selection model (M8 in PAML) suggests that ≈ 9% of *Rhox2 *amino acid sites are under positive selection. For *Rhox3*, the test of variable ratios among sites (codons) suggests that no particular codon is under positive selection which is consistent with a potential set of pseudogenes. Using the bayesian approach implemented in Omegamap (see methods) similar regions and codons identified with PAML analyses above were likely to be subject to positive selection (Figure 4C, D). Very different codons appear to be under positive selection in the two genes. For example, the extreme 5' coding portion of the *Rhox2 *appears to be under positive selection wheras the same region appears to be highly constrained in *Rhox4*. The complete lack of overlap in positive selection between *Rhox2 *and *Rhox4 *suggests that these two genes families have diverged to perform different functions. There is little evidence of recombination between paralogues (ie. Between *Rhox2 *and *Rhox4*) as these genes are very different from each other. Regardless, it appears that the possible presence of gene conversion or recombination has not inhibited our ability to detect positive selection on some codons at the *Rhox *cluster.

### *Rhox2 *has a comparable function to *Rhox4 *in ES cells

Given the complexity of this genomic region, it is not possible to specifically delete *Rhox2*, *Rhox3 *or *Rhox4 *from the genome by standard homologous recombination technology. A more complex genome engineering approach would have to be employed to delete the whole duplicated region and subsequent transgenic rescue approaches performed to assign specific functions (if any) to *Rhox2, Rhox3 or Rhox4*. As an alternative, and more immediate approach we have used an overexpression strategy in an attempt to compare the function of the different *Rhox *genes.

**Figure 5:**
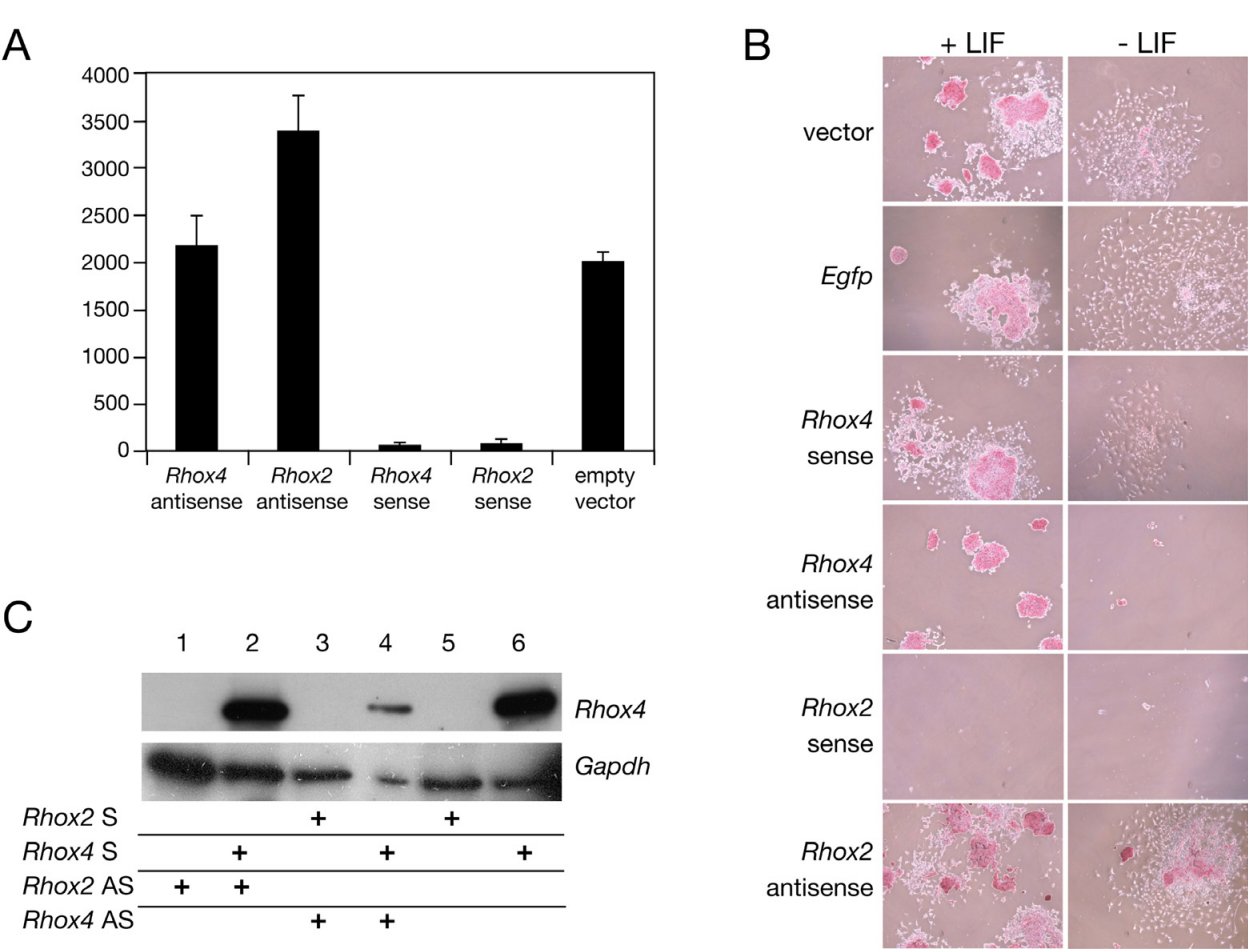
A. Total number of puromycin-resistant colonies generated after supertransfection of episomal vectors expressing *Rhox2*, *Rhox4*, anti-sense *Rhox2*, anti-sense *Rhox4 *or a control empty vector. Mann Whitney test revealed significant differences between control and *Rhox2 *sense (P < 0.03) and control and *Rhox4 *sense (P < 0.03). B. Alkaline phosphatase stained ES cell cultures after supertransfection with either an empty episomal vector (vector) or an *Egfp*-, *Rhox4*-, antisense *Rhox4*, *Rhox2*- or antisense *Rhox2*- expressing episomal vector. Transfected cells were selected in puromycin for 6 days and subsequently replating in the presence (+) and absence (-) of LIF. C. Western blot analysis of COS7 cells transiently co-transfected with vectors expressing sense (S) *Rhox2 *(lane 3 and 5) or *Rhox4 *(lanes 2, 4 and 6) and vectors expressing anti-sense (AS) *Rhox2 *(lanes 1 and 2) or anti-sense *Rhox4 *(lanes 3 and 4) showing efficient knockdown of RHOX4 protein product in the presence of anti-sense *Rhox4 *(lane 4) but not anti-sense *Rhox2 *(lane2). Blots were stripped and re-probed with GAPDH to ensure equivalent loading.

We had previously identified *Rhox4 *(*Ehox*), as a crucial factor in the early stages of ES cell differentiation [12]. We identified *Rhox2 *as the most closely related gene to *Rhox4 *within the *Rhox *cluster, so we were interested to assess whether it had a comparable function. We therefore used the same episomal expression system [18,19] to directly compare the function of these two genes in this system. *Rhox2 *and *Rhox4 *are expressed at comparable levels in undifferentiated ES cells and are downregulated as ES cells initiate differentiation in the absence of Leukaemia Inhibitory Factor (LIF) [12] (data not shown). We directly compared the effects of altering the level of expression of *Rhox2 *and *Rhox4 *on the self-renewal and differentiation potential of ES cells both in the presence and absence of LIF. E14/T ES cells were transfected with constructs expressing RHOX2A or RHOX4B, cultures were selected in puromycin in the presence of LIF and the numbers of resistant colonies were counted six days later (Figure 5A). Flow cytometry of enhanced green fluorescent protein (eGFP) transfected control cultures and the expression levels of *Rhox2 *anti-sense transcripts in ES cells transfected with *Rhox2 *anti-sense expression constructs demonstrated a very high transfection efficiency [see Additional file 4]. We confirmed our previous findings that over-expression of RHOX4B was incompatible with the maintenance of undifferentiated ES cells [12] as indicated by the significant reduction in the number of puromycin resistant colonies. Over-expression of RHOX2A had a similar effect with virtually no colonies surviving after puromycin selection. However, we were able to maintain a pool of RHOX4B-over-expressing ES cells upon replating in LIF [12] (Figure 5B) whereas it proved impossible to maintain a line of ES cells over-expressing RHOX2A (Figure 5B). This difference could either reflect slight functional differences between RHOX2A and 4B or by the differences in expression levels that are achieved using the Internal Ribosomal Entry Site- Puromycin resistance (IRES-PURO) construct. We can conclude that expression of RHOX2A or RHOX4B at a high level is incompatible with an undifferentiated ES cell phenotype possibly driving them into a differentiated cell type that has a significantly reduced viability and/or clonogenic potential.

We used constructs carrying full-length anti-sense sequence of *Rhox2A *and *Rhox4B *that have the potential to block expression of endogenous transcripts. We have observed successful knockdown of RHOX4B protein when anti-sense *Rhox4B *was co-transfected in COS7 cells [12] and we see no reduction in RHOX4B protein when anti-sense *Rhox2A *is co-transfected with the *Rhox4B-*expressing vector (Figure 5C). Furthermore, the anti-RHOX4 antibody does not detect a band in COS7 cells transfected with *Rhox2A*-expressing plasmid (Figure 5C) so taken together these data suggest that this strategy can be effective and specific. Given the similarity between paralogues of each gene we predict that this type of anti-sense approach could knockdown all paralogues of the gene under test.

*Rhox4 *anti-sense expression resulted in a comparable number of colonies compared to control transfections, whereas *Rhox2 *anti-sense expression resulted in a slight increase in the number of puromycin resistant colonies (Figure 5A). This could indicate that blocking RHOX2 expression using the full-length anti-sense construct conferred a survival advantage on undifferentiated ES cells and contributed to them maintaining their undifferentiated state and self-renewal capacity. The phenotype of alkaline phosphatase-stained colonies after puromycin selection and subsequent re-plating in the absence of LIF further substantiated this finding (Figure 5B). We had previously shown (and confirm here) that when LIF is withdrawn from ES cells expressing anti-sense *Rhox4*, small, undifferentiated colonies were detected but no differentiated cells were observed (Figure 5B). This suggested that blocking the expression of RHOX4 inhibits ES cell differentiation even in the absence of LIF. In contrast, when LIF is withdrawn from anti-sense *Rhox2 *expressing cells a proportion of cells remain undifferentiated but we also observe a significant number of differentiated cells. Thus inhibiting expression of RHOX2 and RHOX4 apparently results in a partial or complete block in differentiation, respectively. A block in differentiation is also apparent in anti-sense *Rhox4 *expressing cells when cultured in the presence of LIF, where all colonies are alkaline phosphatase positive (stem cell) with no spontaneous differentiation observed (Figure 5B). Many spontaneously differentiating cells are observed when anti-sense *Rhox2 *expressing cells are cultured in LIF (Figure 5B), a finding that is consistent with the idea that blocking RHOX2 only partially blocks differentiation. This qualitative difference in the degree of differentiation may reflect a functional difference between RHOX2 and RHOX4 in ES cells. Alternatively, this observation could be explained by differences in the levels of sense and anti-sense expression. We can conclude however, that the levels of expression of both RHOX2 and 4 play a role in the early stages of ES cell differentiation *in vitro*.

## Discussion

We have identified extensive tandem duplications of *Rhox2*, *3 *and *4 *on the mouse X chromosome resulting in a total of 32 *Rhox *genes of which all but three are predicted to produce functional proteins. The *Rhox *cluster therefore, has more genes than any other homeobox cluster identified in any species. Moreover, the degree of similarity between the genomic regions and molecular evolutionary analysis of *Rhox2*, *3 *and *4 *suggests that this represents the most recent homeobox gene duplication identified to date. Despite such a recent duplication event, *Rhox2 *and *4 *paralogues show evidence of positive selection in their evolutionary history suggesting a potential function for these genes. We have also identified a role for *Rhox2 *and *Rhox4 *in the maintenance and differentiation of ES cells supporting the biologically importance of this duplication.

### A mouse specific duplication of *Rhox2*, *3 *and *4*

The lack of sequence divergence between each 40 kb duplication unit and the *dS *values for *Rhox2*, *3 *and *4 *imply that the duplication events are relatively young. We predict that the duplications occurred after the divergence of mouse and rat. The alignment of over 70,000 exons common to mouse and rat predicts a synonymous substitution rate of 0.17 [16] over 10-fold greater than the *Rhox2 *paralogues with a *dS *value of 0.0122 and 100-fold greater for the *Rhox4 *paralogues at 0.001. Obviously, the *dS *values are an average for all *Rhox2*, *3 *and *4 *paralogues and do not reflect the sequential nature of the duplication events. Nevertheless, assuming that mouse and rat diverged 16 million years ago [20,21], a constant molecular clock would result in the relative age of the *Rhox *duplicates being less than one tenth the time split between mouse and rat or approximately <1.6 million years. However, the *dS *ratio is not an accurate measure of time as unequal cross-over and gene conversion events can act to alter *dS *values. This may be particularly relevant in a region showing such repetitive similarity.

*Mus spretus *and *Mus domesticus *are predicted to have diverged from *Mus musculus *approximately three million and one million years ago respectively [22]. The presence of multiple *Rhox4 *copies in *Mus spretus *suggests that the duplication events were well underway before the divergence of these species 3 million years ago. Preliminary Southern blot analysis using mouse specific *Rhox4 *probes suggest that, at most, two copies of *Rhox4 *exist in rat (data not shown). Moreover, we screened the rat genome (RGSC v3.4) with the highly conserved exon 3 sequence of *Rhox2*, *3 *and *4 *to see if we could identify different paralogues of these genes as evidence for the presence of these duplications in rat. We identified a single homologue of *Rhox4 *on the rat X chromosome (95% similarity) but none for *Rhox2 *or *3*. By comparison the same screen in mouse identifies multiple copies with different chromosomal coordinates. Although the synonymous substitution rate and absence of *Rhox *paralogues in the rat do not definitively show the absence of *Rhox *duplicates in rat these data support our contention that the duplications are murine specific.

### Molecular evolution of the *Rhox *cluster

The molecular evolution analysis of all paralogues, individual paralogues and individual codons within paralogues suggests that both *Rhox2 *and *Rhox4 *have different degrees of diversifying selection in their evolutionary history whereas *Rhox3 *is evolving neutrally. An extensive analysis of single gene duplicates from a number of species reveals that duplicates typically undergo a phase of relaxed selection or even accelerated evolution at replacement sites and progressively become more constrained as they age [23]. Although we report a multiple duplication event, our data is consistent with this observation. The differences observed between the evolutionary profiles of *Rhox2*, *3 *and *4 *could reflect a number of evolutionary and functional differences. *Rhox2*, *3 *and *4 *are expressed at different stages in the development of the testes and ES cell differentiation and *Rhox4 *is uniquely expressed in the developing and adult thymus [3,24]. We predict that such differing biological functions for *Rhox2*, *3 *and *4 *will put different evolutionary constraints on these genes resulting in different evolutionary outcomes.

Despite the relatively young age of these duplications these data supports divergent selection pressures on a subset of *Rhox2 *and *Rhox4 *paralogues that is driving functional change. This is at odds with many other transcription factors, including *Hox *genes, which typically show strong evolutionary constraint [25]. However, it is consistent with a function for *Rhox *genes in reproduction. Sexual conflict, sexual selection and sperm competition are predicted to exert a strong selective pressure driving the rapid evolution of reproductive genes including transcription factors [26,27]. For *Rhox2 *we identified two lineages that show significant diversifying selection in their evolutionary history. This data is indicative of a degree of neo-functionalisation for *Rhox2A, D *and *E *relative to the other *Rhox2 *paralogues. Within *Rhox2 *and *4*, individual codons have been identified which show evidence of positive selection. Of interest are *Rhox2*(132) and *Rhox4*(170)which translate to non-conservative substitutions within the homeobox region of these genes. Mutations within homeodomains are significant as subtle changes in DNA binding affinity could have important biological affects due to the alteration of downstream gene activation or repression. Consequently, there exists important constraint on homeodomain evolution with *Rhox5 *providing one of the few examples of positive selection within this region [27,28].

One surprising feature of the molecular evolution of *Rhox2*, *3 *and *4 *is the marked differences in the synonymous substitution rates. Differences in base composition, variable mutation rate and gene conversion are all mechanism by which the synonymous substitution rates could vary between genes. Studies in *E*.*coli *and *S. cerevisiae *suggest a relationship between gene expression levels and mutation rates [29,30]. Although its significance in mammals is unknown, *Rhox2 *is expressed 5-fold higher than *Rhox4 *during testis development [3]. Moreover, if the transcriptional control elements of each gene are preserved between duplication units then so will the mechanism for variable *dS *values between genes. Gene conversion could be a factor in suppressing sequence variation in *Rhox3 *and *Rhox4 *paralogues but not *Rhox2 *although, again, this would have to be consistent over all duplication units to maintain the discrepancy in *dS *values. Regardless of mechanism, *Rhox2 *paralogues show significantly more sequence variation than *Rhox3 *and *4*, which may affect the evolutionary trajectories of these genes. Regardless of whether gene conversion is contributing to the evolution of these genes, it is unlikely to affect our inferences of adaptation at this region [31].

### *Rhox *function in ES cells

We provide experimental evidence to show that at least two genes (*Rhox2 *and *Rhox4*) within the duplicated region of the *Rhox *cluster play a role in the maintenance and early differentiation of ES cells. Although we used paralogues RHOX2A and RHOX4B in these studies, the similarity between the *Rhox2 *and *Rhox4 *paralogues would predict that these other paralogues would have comparable effects. Both genes are expressed in undifferentiated ES cells so we tested the effects of altering the levels of expression of RHOX2 and RHOX4 on the undifferentiated phenotype. This strategy has revealed other homeobox-containing genes, *Oct4 *and *Nanog *as key stem cell regulators [19,32]. Increases in *Oct4 *expression levels in ES cells induces differentiation in a manner that is dominant over the suppressive effects of LIF and overexpression of *Nanog *has been shown to drive self-renewal of ES cells in the absence of LIF. We show that artificially high levels of RHOX2 or RHOX4 are incompatible with an undifferentiated ES cell phenotype either because such high levels of these proteins affect the viability of undifferentiated ES cells or because they override the effects of LIF and drive undifferentiated ES cells into a differentiated state that cannot survive. We favour the latter explanation because when we reduce the levels of RHOX4, differentiation of ES cells appears to be blocked. Reduction of RHOX2 protein increases the numbers of self-renewing colonies in the presence of LIF and, in the absence of LIF, blocking either RHOX2 or RHOX4 results in is a persistence of self-renewing stem cells that are not observed in control cultures. As mentioned, we predict that the anti-sense approach we have used to disrupt *Rhox *gene function is likely to affect all paralogues. A siRNA approach could extend these studies to assess the function of the individual paralogues.

Although ES cells have proven invaluable as a research tool they are not considered to be entirely equivalent to the inner cell mass tissue from which they are derived [33]. Consequently, relating the function of *Rhox *proteins in the *in vitro *ES cells system to any evolutionary analysis is inappropriate. However, RHOX4 is expressed in trophoblast stem (TS) cells *in vitro *and *in vivo *supporting a function for *Rhox4 *in the stem cell compartment of developing placenta that would provide selective pressure for evolutionary change [3,24]. Consequently, the analysis of *Rhox *function in TS cells would be more applicable to molecular evolutionary data. For example, over-expression of different *Rhox *paralogues in TS cell cultures may help identify whether positive selection of certain paralogues or codons is associated with differences in downstream gene expression changes.

Our data suggest that the expression levels of *Rhox2 *and *4 *may be crucial to their function in ES cells and this phenomenon may be applicable to other cell types in which they are expressed. Gene duplication is used by a number of genes, for example rRNA and histones, as a means of driving high-level gene expression. The possibility arises that the number of *Rhox2*, *3 *and *4 *paralogues is linked to the expression levels of these genes. The modulation of *Rhox *gene expression levels in ES cells and the consequent affects on ES biology will begin to address this issue.

Our studies reveal the crucial role for this duplicated region of the *Rhox *cluster in murine ES cell biology. It is well documented that ES cells derived from mouse, rat and human exhibit markedly different characteristics in terms of their growth requirements and developmental potential [34,35]. The mouse specific duplication of *Rhox2*, *3 *and *4 *and the rodent specific *Rhox *cluster are obvious candidates for mediating these species differences.

## Conclusion

The *Rhox *cluster therefore appears a very plastic region of the rodent genome with the mouse containing twenty more *Rhox *genes than rat and rat containing ten more *Rhox *genes than human. Expression in the reproductive tissues and a function for *Rhox5 *in male fertility has lead to the hypothesis that the rodent specific cluster may, in part, mediate the higher reproductive capacity of rodents relative to humans. The expanded *Rhox *cluster in the mouse provides a large number of substrates for the generation of evolutionary novelties. This is of particular interest as it has been postulated that both gene duplication and the rapid evolution of reproductive proteins are an important mechanism in speciation [23,26].

## Methods

### Genome sequence analysis

The genomic sequence used for this study is from the NCBI m33 mouse assembly (freeze May 27, 2004, strain C57BL/6J). The dotplot was created by comparing the first duplication unit ("A") to the entire *rhox *cluster region. We used Advanced Pipmaker on the Pipmaker website [36] with the Dotplot and single coverage option. Repeat content has been determined using RepeatMasker. Presence of LINE fragments larger than 500 bp have been annotated in the dot plot. Similarity between repeat units was calculated by first removing repeat elements insertions and small rearrangements. Sequences were subsequently compared using the BLAST algorithm and an overall percentage similarity match calculated.

### Southern blot

Southern blotting was carried out as previously described [37]. Briefly, genomic DNA from CGR8 ES cells was digested with Asp718 and HindIII and hybridised to a 208 bp probe spanning *Rhox4 *exon1 and intron1. *Mus domesticus *and *Mus spretus *genomic DNA was digested with HindIII and hybridised to a 370 bp *Rhox4 *exon 2 probe.

### Sequence alignment and expression analysis

The individual paralogues of *Rhox2*, *3 *and *4 *were assembled from the genomic sequence (see above). Alignments were carried out using the ClustalW algorithm within the MegAlign program of DNAstar [38]. The individual profiles of each paralogue were determined by selecting 10 nucleotides that gave a unique profile. RNA was isolated from CGR8 ES cells and placenta and RT-PCR was performed as described previously [12] using the primers *Rhox2*-5' GGAATAAGGACTTCCACGGCTTTACA and *Rhox2*-3' AACTGTGTTGTAACAGGGCTTTGGCGGC to amplify *Rhox2A*-*H *and *Rhox4*-5' CGACTCAGAATCTGCTGGGG *Rhox4*-3' CAGGGGTCTGCACGTGGCTC to amplify *Rhox4A*-*H*. PCR products were TA cloned (Invitrogen) and sequenced. The BLAST algorithm [39] was used to search the mouse subset database of expressed sequences using the full length *Rhox2A *or *Rhox4A *sequence (most recently analysed July 2005) and positive hits compared to the unique nucleotide profile for each paralogue of *Rhox2 *and *Rhox4*. Each unique nucleotide profile for *Rhox2 *and *4 *has a minimum of 3 different nucleotides between paralogues with the exception of *Rhox2B *and *E *(2 nucleotides different), *Rhox2D *and *G *(1 nucleotide difference) and *Rhox4A *and *C *(2 differences). Misidentification of individual paralogues due to sequencing errors would require a minimum of 3 independent sequencing errors in these positions and is therefore extremely unlikely.

### Molecular evolution

Sequences were aligned using ClustalX Maximum likelihood estimates of *dN/dS *(the parameter) for each lineage in the phylogenies (see Figure 3) were derived by a method employing different evolutionary models, using the CODEML program of PAML Version 3.0 b [14,15,40,41]. Investigation of evolutionary rates between lineages was carried out using alternative likelihood models, one with a single dN/dS ratio (M0, one-ratio) [42] estimated for all branches and another that allowed independent dN/dS ratios for each branch (FR, free-ratio) [15]. Statistical testing of differences between the different models [14,15,40] was carried out using twice the log likelihood difference (2*l*) which conforms to a chi-square distribution (the Likelihood Ratio test, LRT), with the degrees of freedom based on the difference between the number of parameters estimated from the models. Sequences Rhox3B, Rhox3D and Rhox3G have stop codons at intermediate positions in the coding regions. The third nucleotide position of these codons has been adjusted as undetermined in order to allow us to perform *dN/dS *analyses. The posterior p-values for estimates of dN/dS for each codon (using model M8) are also shown and were inferred using PAML.

To infer selection across codons within the *Rhox *clusters, we also used a new method implemented in omegamap [17] for estimating the selection parameter from a sample of potentially recombining gene sequences. Uncertainty in the evolutionary history was taken into account using a coalescent-based approximate (PAC) likelihood. Variation was modelled as a block-like structure with a variable number of blocks. We averaged over the number and position of the blocks using reversible-jump MCMC to obtain the posterior distribution of the parameters (specifically *dN/dS *in this case). We output the *dN/dS *value for each codon as well as its posterior p-value. Posterior values greater than 0.95 (blue line) indicate likely codons subject to positive selection (*dN/dS *> 1).

### ES cell cultures

ES cells were cultured as described previously [12]. E14/T ES cells are transgenic for polyomavirus large T and can be supertransfected with a second plasmid containing polyoma ori. The second plasmid is maintained at high copy number as an episome and achieves high levels of expression of the target gene [18,19]. This second plasmid contains either *Rhox2 *or 4 in sense or antisense orientations on a bicistronic expression cassette driven by the CAG promoter with puromycin resistance coupled to expression via an IRES element. E14/T were electroporated with the expression plasmids and selected in 0.75 – 1.0 ug/ml puromycin (Sigma) for 6 days in GMEM +LIF. To assess the effects of withdrawal of LIF, cultures were trypsinised and 10^4 ^cells were replated into 6 well plates in the presence of puromycin. Cultures were stained for alkaline phosphatase (Sigma leukocyte kit) and colonies were scored as undifferentiated (alkaline phosphatase positive), differentiated (alkaline phosphatase negative) or mixed (partially alkaline phosphatase positive).

### COS7 cell transfection

COS7 cells were plated into 6 well plates and transfected with 2 μg of the appropriate plasmid using Fugene accordingly to manufacturers instructions (Roche). Cells were lysed after 2 days in 2 × lameli sample buffer.

## Authors' contributions

MJ carried out the ES cell experiments, genomic and gene sequence analysis and participated in the design of the study and helped to draft the manuscript. AJW participated in the Southern Blot analysis, analysed the genomic and gene sequence data, participated in the experimental design and drafted the manuscript. PG carried out the analysis of genomic sequence using Pipmaker and BLAST. DG carried out the Southern blot analysis and genome sequence analysis. JD participated in the ES cell experiments. GJG conceived of part of the study. JK, FP and PA conceived of, designed and performed the evolutionary analysis. LMF conceived of the study, participated in its design and coordination and helped to draft the manuscript.

## Supplementary Material

Additional file 1 Table showing the intron/exon sizes in base pairs for each paralogue of *Rhox2, 3 & 4*. Click here for file

Additional file 2 ClustalW alignment of predicted cDNAs for *Rhox2A-H*, *Rhox3A-H *and *Rhox4A-H*. Click here for file

Additional file 3 Unique nucleotide profile of predicted *Rhox3A-H *transcripts and Clustal W alignment of predicted amino acid sequence from *Rhox3A-H*. Click here for file

Additional file 4 Analysis of transfection efficiency of E14/T ES cells with eGFP control and *Rhox2 *anti-sense constructs. Click here for file

## Figures and Tables

**Table 1:** Calculation of *dN*,*dS *and ω values for all *Rhox2*,*3 *and *4 *paralogues.

Gene	*dN*	*dS*	ω
*Rhox2*(*A*-*H*)	0.010	0.0122	0.82
*Rhox3*(*A*-*H*)	0.002	0.004	0.5
*Rhox4*(*A*-*H*)	0.004	0.001	4.00
