# Supplementary material for: A murine specific expansion of the Rhox cluster involved in embryonic stem cell biology is under natural selection
Source: BMC Genomics. 2006 Aug 17;7:212. doi: 10.1186/1471-2164-7-212 (PMC1562416; doi:10.1186/1471-2164-7-212)
Supplement: Additional file 1 — Table showing the intron/exon sizes in base pairs for each paralogue of Rhox2, 3 & 4. [file 1471-2164-7-212-S1.doc]

# Rhox2

|  | **A** | **B** | **C** | **D** | **E** | **F** | **G** | **H** |
| --- | --- | --- | --- | --- | --- | --- | --- | --- |
| **exon1*** | 96 | 96 | 96 | 96 | 96 | 96 | 96 | 96 |
| **intron1** | 162 | 162 | 162 | 162 | 162 | 162 | 162 | 162 |
| **exon2** | 412 | 412 | 412 | 412 | 412 | 412 | 412 | 412 |
| **intron2** | 1515 | 1540 | 1528 | 1556 | 8153 | 1545 | 1583 | 1543 |
| **exon3** | 46 | 46 | 46 | 46 | 46 | 46 | 46 | 46 |
| **intron3** | 2239 | 2239 | 2248 | 1792 | 2248 | 2253 | 1814 | 1797 |
| **exon4*** | 226 | 226 | 226 | 226 | 226 | 226 | 226 | 226 |

## Rhox3

|  | **A** | **B** | **C** | **D** | **E** | **F** | **G** | **H** |
| --- | --- | --- | --- | --- | --- | --- | --- | --- |
| **exon1*** | 175 | 175 | 175 | 175 | 175 | 175 | 175 | 175 |
| **intron1** | 250 | 250 | 250 | 250 | 250 | 250 | 250 | 250 |
| **exon2** | 370 | 370 | 370 | 370 | 370 | 370 | 370 | 370 |
| **intron2** | 1876 | 1875 | 1869 | 1875 | 1877 | 1873 | 2720 | 1872 |
| **exon3** | 46 | 46 | 46 | 46 | 46 | 46 | 46 | 46 |
| **intron3** | 1147 | 1148 | 1145 | 1190 | 1148 | 1189 | 1149 | 1149 |
| **exon4*** | 236 | 236 | 236 | 236 | 236 | 236 | 236 | 236 |

## Rhox4

|  | **A** | **B** | **C** | **D** | **E** | **F** | **G** | **H** |
| --- | --- | --- | --- | --- | --- | --- | --- | --- |
| **exon1*** | 118 | 118 | 118 | 118 | 118 | - | 118 | 118 |
| **intron1** | 190 | 190 | 193 | 190 | 190 | - | 190 | 194 |
| **exon2** | 406 | 406 | 406 | 406 | 406 | - | 406 | 406 |
| **intron2** | 2407 | 2400 | 2419 | 2418 | 2393 | - | 2400 | 2367 |
| **exon3** | 46 | 46 | 46 | 46 | 46 | - | 46 | 46 |
| **intron3** | 1273 | 1309 | 1273 | 1274 | 1310 | - | 1273 | 1288 |
| **exon4*** | 236 | 236 | 236 | 236 | 237 | - | 236 | 236 |

### Additional File 1

Each table shows the intron / exon sizes in base pairs for each paralogue of *rhox2*-*4*

* the size of exon1 and exon4 have not been determined empirically. The sizes provided are calculated from an arbitrary position relative to the published translational start site for exon1 and the 5’ splice acceptor of exon4 for exon4.
