# Supplementary material for: A murine specific expansion of the Rhox cluster involved in embryonic stem cell biology is under natural selection
Source: BMC Genomics. 2006 Aug 17;7:212. doi: 10.1186/1471-2164-7-212 (PMC1562416; doi:10.1186/1471-2164-7-212)
Supplement: Additional file 2 — ClustalW alignment of predicted cDNAs for Rhox2A-H, Rhox3A-H and Rhox4A-H. [file 1471-2164-7-212-S2.doc]

***rhox2***

***rhox2A*** TGAATAAGGACTTCCACGGCTTTACAGACATGGAGCGACAAAGCGTCAATTACAAGCTTGATGTGGGACCTGAGG 75

***rhox2B*** TGAATAAGGACTTCCACGGCTTTACAGACATGGAGCGACAAAGCGTCAATTACAAGCTTGATGTGGGACCTGAGG 75

***rhox2C*** TGAATAAGGACTTCCACGGCTTTACAGACATGGAGCGACAAAGCGTCAATTACAAGCTTGATGTGGGACCTGAGG 75

***rhox2D*** TGAATAACGACTTCCACGGCTTTACAGACATGGAGCAACAAAGCATCAATTACAAGCTTGATGTGGGACCTGAGG 75

***rhox2E*** TGAATAAGGACTTCCACGGCTTTACAGACATGGAGCGACAAAGCGTCAATTACAAGCTTGATGTGGGACCTGAGG 75

***rhox2F*** TGAATAAGGACTTCCACGGCTTTACAGACATGGAGCGTCAAAGCATCAATTACCTGCTTGATGTGGGACCTGAGG 75

***rhox2G*** TGAATAACGACTTCCACGGCTTTACAGACATGGAGCGACAAAGCATCAATTACAAGCTTGATGTGGGACCTGAGG 75

***rhox2H*** TGAATAACGACTTCCACGGCTTTACAGACATGGAGAAAAGAAGCATCAATTACCTGCTTGATGTGGGACCTGAGG 75

***rhox2A*** AGGATGAGGAAAATGCGAATGGTGTAAAGACTCTGATGGTCTTGCTGGCTGGAGAGGGAAGAAATGAGGGAGAGA 150

***rhox2B*** AGGATGAGGAAAATGCGAATGGTATAAAGACTCTGATGGTCTTGCTGGCTGGAGAGGGAAGAAATGAGGGAGAGA 150

***rhox2C*** AGGATGAGGAAAATGCGAATGGTATAAAGACTCTGATGGTGTTGCTGGCTGGAGAGGGAAGAAACGAGGGAGAGA 150

***rhox2D*** AGGATGAGGAAAATGCGAATGGTGTAAAGACTCTGATGGTGTTGCTGGCTGGAGAGGGAAGAAACGAGGGAGAGA 150

***rhox2E*** AGGATGAGGAAAATGCGAATGGTGTAAAGACTCTGATGGTCTTGCTGGCTGGAGAGGGAAGAAACGAGGGAGAGA 150

***rhox2F*** AGGATGAGGAAAATGCGAATGGTGTAAAGACTCTGATGGTCTTGCTGGCTGGAGACGGAAGAAACGAGGGAGAGA 150

***rhox2G*** AGGATGAGGAAAATGCGAATGGTATAAAGACTCTGATGGTGTTGCTGGCTGGAGAGGGAAGAAACGAGGGAGAGA 150

***rhox2H*** AGGATGAGGAAAATGCGAATGGTGTAAAGACTCTGATGGTCTTGCTGGCTGGAGAGGGAAGAAACGAGGGAGAGA 150

***rhox2A*** GTGGACGGGGCCTGCCTGGGTCGGGAGCCTCAGCAGCGGAAGGATACAGAGCAGGAGAAATAAGTGCAGGTGGGC 225

***rhox2B*** GTGGACCGGGCCTGCCTGGGTCGGGAGCCTCAGCAGCGGAAGGATACAGAGCAGGAGAAATAAGTGCAGGTGGGC 225

***rhox2C*** GTGGACGGGGCCTGCCTGGGTCGGGAGCCTCAGCAGCGGAAGGATACAGAGCAGGAGAATTAAGTGCAGGTGGGC 225

***rhox2D*** GTGGACCGGGCCTGCCTGGGTCGGGAGTCTCAGCAGCGGAAGGATACAGAGCAGGAGAATTAAGTGCAGGTGGGC 225

***rhox2E*** GTGGACGGGGCCTGCCTGGGTCGGGAGCCTCAGCAGCGGAAGGATACAGAGCAGGAGAAATAAGTGCAGGTGGGC 225

***rhox2F*** GTGGACGGGGCCTGCCTGGGTCAGGAGCCTCAGCAGCGGAAGGATACAGAGCAGGAGAAATAAGTGCAGGTGGGC 225

***rhox2G*** GTGGACGGGGCCTGCCTGGGTCGGGAGTCTCAGCAGCGGAAGGATACAGAGCAGGAGAATTAAGTGCAGGTGGGC 225

***rhox2H*** GTGGACGGGGCCTGCCTGGGTCGGGAGTCTCAGCAGCGGAAGGATACAGAGCAGGAGAATTAAGTGCAGGTGGGC 225

***rhox2A*** CTGCTGCGCCAGTAGCCGACCTCATGGATAACAGCAACCAAGAGGACCTTGGTGCCACTGGCTGTGACCAGGAGA 300

***rhox2B*** CTGCTGCGCCAGTAGCCGACCTCATGGATAACAGCAACCAAGAGGACCTTGGTGCCACTGGCTGTGACCAGGAGA 300

***rhox2C*** TTGCTGCGCCAGTAGCCGACCTCATGGATAACAGCAACCAAGAGGACCTTGGTGCCACTGGCTGTGACCAGGAGA 300

***rhox2D*** TTGCTTCGCCAGTAGCCGACCTCATGGATAAGAGCAACCAAGAGGACCTTAGTGCCACTGGCTGTGCCCAGGAGA 300

***rhox2E*** CTGCTGCGCCAGTAGCCGACCTCATGGATAACAGCAACCAAGAGGACCTTGGTGCCACTGGCTGTGACCAGGAGA 300

***rhox2F*** CTGCTGCGCAAGTAGCCGACCTCATGGATGACAGCAACCAAGAGGACCTTGGTGCCACTGCCTGTGACCAGGAGA 300

***rhox2G*** TTGCTGCGCCAGTAGCCGACCTCATGGATAACAACAACCAAGAGGACCTTGGTGCCACTGGCTGTGCCCAGGAGA 300

***rhox2H*** CGGCTGCGCCTGTAGCAGGCCTCATGGATAACAGCAACCAAGAGGACCTTAGTGCCACTGGCTGTGCCCAGGAGA 300

***rhox2A*** AGGAGAAGCAGCCAGAGGAGCCAGTCCCTGATTCCATGGGAGATTTGGAAAATGTAAAGCGTGTGTCCGGGCCGT 375

***rhox2B*** AGGAGAAGCAGCCAGAGGAGCCAGTCCCTGATTCCATGGGAGATTTGGAAAATGTAAAGCATGTGTCCGGGCCGT 375

***rhox2C*** AGGAGAAGCAGCCAGAGGAGCCAGTCCCTGATTCCATGGGAGATTTGGAAAATGTAAAGCGTGTGTCCGGGCCGT 375

***rhox2D*** AGGAGAAGCAGCCAGAGGAGCCAGTCCCTGATTCCATGGGGGATTTGGAAAATGTAAAGCCTATGTCAGGGCCGT 375

***rhox2E*** AGGAGAAGCAGCCAGAGGAGCCAGTCCCTGATTCCATGGGAGATTTGGAAAATGTAAAGCGTGTGTCCGGGCCGT 375

***rhox2F*** AGGAGAAGCAGCCAGAGGAGCCAGTCCCTGATTCCATGGGAGATTTGGAAAATGTAAAGCGTGTGTCCGGGCCGT 375

***rhox2G*** AGGAGAAGCAGCCAGAGGAGCCAGTCCCTGATTCCATGGGGGATTTGGAAAATGTAAAGCCTATGTCAGGGCCGT 375

***rhox2H*** AGGAGACGCAGCCAGAGGAGCCAGTCCCTGATTCCATGGGGGATTTGGAAAATGTAAAGCCTATGTCAGGGCCGT 375

***rhox2A*** GGTCCACTGTTAATCCTGTGAGAGTGTTGGTGCCCGAATTCCGCCACGGTTGGCAACAGAGCTTCAATGTGCTGC 450

***rhox2B*** GGTCCACTGTTAATCCTGTGAGAGTGTTGGTGCCCAAATTCCGCCACGGTTGGCAACAGAGCTTCAATGTGCTGC 450

***rhox2C*** GGTCCACTGTTAATCCTGTGAGAGTGTTGGTGCCCAAATTCCGCCACGGTTGGCAACAGAGCTTCAATGTGCTGC 450

***rhox2D*** GGTCCACTGTTAATCCTGTGAGAGTGTTGGTGCCCGAATTCCGCTACAGTTGGCAACAGAGCTTCAATGTGCTGC 450

***rhox2E*** GGTCCACTGTTAATCCTGTGAGAGTGTTGGTGCCCGAATTCCGCCACGGTTGGCAACAGAGCTTCAATGTGCTGC 450

***rhox2F*** GGTCCACTGTTAATCCTGTGAGAGTGTTGGTGCCCAAATTCCGGCACCTTTGGCGACACAGCTTCAATGTGCTGC 450

***rhox2G*** GGTCCACTGTTAATCCTGTGAGAGTGTTGGTGCCTGAATTCCTTCACGGTTGGCAACAGAGCTTCAATGTGCTGC 450

***rhox2H*** GGTCCACTGTTAATCCTGTGAGAGTGTTGGTGCCCAAATTCCGGCACCTTTGGCGACACAACTTCAATGTGCTGC 450

***rhox2A*** AACTACAAGAGCTGGAGAGCATCTTCCAGTGCAATCACTACATCAGCACTAAGGAGGCAAATCGCCTGGCAAGAT 525

***rhox2B*** AACTACAAGAGCTGGAGAGCATCTTCCAGTGCAATCACTACATCAGCACTAAGGAGGCAAATCGCCTGGCAAGAT 525

***rhox2C*** AACTACAAGAGCTGGAGAGCATCTTCCAGTGCAATCACTACATCAGCACTAAGGAGGCAAATCGCCTGGCAAGAT 525

***rhox2D*** AACTACAAGAGCTGGAGAGCATCTTCCAGTGCAATCAATACATCAGCACTACAGAGGCAAAACGTCTGGCAAAAT 525

***rhox2E*** AACTACAAGAGCTGGAGAGCATCTTCCAGTGCAATCACTACATCAGCACTAAGGAGGCAAATCGCCTGGCAAGAT 525

***rhox2F*** AACTACAAGAGCTGGAGAGCATCTTCCAGTGCAATCACTACATCAGCACTAAGGAGGCAAATCGCCTGGCAAGAT 525

***rhox2G*** AACTACAAGAGCTGGAGAGCATCTTCCAGTGCAATCACTACATCAGCACTACAGAGGCAAAGTGTCTGGCAAGAT 525

***rhox2H*** AACTACAAGAGCTGGAGAGCATCTTCCAGTGCAATCACTACATCAGCACTACAGAGGAAAATCGCCTGGCAAGAT 525

***rhox2A*** CCATGGGAGTGAGTGAAGCCACAGTGCAGGAATGGTTTTTGAAGAGGAGAGAGAAATACAGGAGTTATAAGAGGC 600

***rhox2B*** CCATGGGAGTGAGTGAAGCCACAGTGCAGGAATGGTTTTTGAAGAGGAGAGAAAAATACAGGAGTTATAAGAGGC 600

***rhox2C*** CCATGGGAGTGAGTGAAGCCACAGTGCAGGAATGGTTTTTGAAGAGGAGAGAAAAATACAGGAGTTATAAGAGGC 600

***rhox2D*** CCATGGGTGTGAGTGAAGCCACAGTGCAGGAATGGTTTTTGAAGAGGAGAGAGAAATACAGGAGTTATAAGAGGC 600

***rhox2E*** CCATGGGAGTGAGTGAAGCCACAGTGCAGGAATGGTTTTTGAAGAGGAGAGAAAAATACAGGAGTTATAAGAGGC 600

***rhox2F*** CCATGGGTGTGAGTGAAGCCACAGTGCAGGAATGGTTTTTGAAGAGGAGAGAAAAATACAGGAGTTATAAGAGGC 600

***rhox2G*** CCATGGGTGTGAGTAAAGCCACAGTGCAGGAATGGTTTTTGAAGAGGAGAGAGAAATACAGGAGTTATAAGAGGC 600

***rhox2H*** CCATGGGTGTGAGTGAAGCCACAGTGCAGGAATGGTTTTTGAAGAGGAGAGAGAAATACAGGAGTTATAAGAGGC 600

***rhox2A*** TGTAAAGGCTCAGAGGTCCTCTTCCTGCATTCCGGAGCATCTCTCTGTGAAGGCTGTGGAGAAGCCCCAGGCAGC 675

***rhox2B*** TGTAAATGCTCAGAGGTCCTCTTCCTGCTTCCCACAGCATCTCTCTGTGAAGGCTATGGAGAAGCCCCAGGGATC 675

***rhox2C*** TGTAAATGCTCAGAGGTCCTCTTCCTGCTTCCCACAGCATCTCTCTGTGAAGGCTATGGAGAAGCCCCAGGGATC 675

***rhox2D*** TGTAAAGGCTCAGAGGTCCTCTTCCTGCATTCCGGAGCATCTCTCTGTGAAGGCTGTGGAGAAGCCCCAGGCAGC 675

***rhox2E*** TGTAAATGCTCAGAGGTCCTCTTCCTGCTTCCCACAGCATCTCTCTGTGAAGGCTATGGAGAAGCCCCAGGGATC 675

***rhox2F*** TGTAAATGCTCAGAGGTCCTCTTCCTGCTTCCCACAGCATCTCTCTGTGAAGGCTATGGAGAAGCCCCAGGGATC 675

***rhox2G*** TGTAAAGGCTCAGAGGTCCTCTTCCTGCATTCCGGAGCATCTCTCTGTGAAGGCTGTGGAGAAGCCCCAGGCAGC 675

***rhox2H*** TGTAAAGGCTCAGAGGTCCTCTTCCTGCATTCCGGAGCATCTCTGTGTGAAGGCTATGGAGAAGCCCCAGGCAGC 675

***rhox2A*** CACCCATGCTCAAGTCACTGTAGACAGACGATGTTGTGCCGCCAAAGCCCTGTTACAACACAGTTATCTCCTCAA 750

***rhox2B*** CACGCTTCCTCAAGGCGCCATAGCCAGACGATGTTTTCCCGCCAAAGTCCTGTTACAACACAGTTATCTCCTCAA 750

***rhox2C*** CACGCTTCCTCAAGGCGCCATAGCCAGACGATGTTTTGCCGCCAAAGCCCTGTTACAACACAGTTATCTCCTCAA 750

***rhox2D*** CACCCATGCTCAAGTCACTGTAGACAGATGATGTTGTGCCGCCAAAGTCCTGTTACAACACAGTTATCTTCTTAA 750

***rhox2E*** CACGCTTCCTCAAGGCGCCATAGCCAGACGATGTTTTCCCGCCAAAGTCCTGTTACAACACAGTTATCTCCTCAA 750

***rhox2F*** CACGCTTCCTCAAGGCGCCATAGCCAGACGATGTTTTCCCGCCAAAGTCCTGTTACAACACAGTTATCTCCTCAA 750

***rhox2G*** CACCCATGCTCAAGTCACTGTAGACAGATGATGTTGTGCCGCCAAAGTCCTGTTACAACACAGTTATCTCCTTAA 750

***rhox2H*** CACCCATGCTCAAGTCACTGTAGACAGACGATGTTGTGCCGCCAAAGCCCTGTTACAACACAGTTATCTCCTCAA 750

***rhox2A*** TACTTGTATTTGCAATAAAGAGCTGAATTC 780

***rhox2B*** TACTTGTATTAGCAATAAAGAGCTGAATTC 780

***rhox2C*** TACTTGTATTAGCAATAAAGAGCTGAATTC 780

***rhox2D*** TACTTGTATTTGCAATAAAGAGCTGAATTC 780

***rhox2E*** TACTTGTATTAGCAATAAAGAGCTGAATTC 780

***rhox2F*** TACTTGTATTAGCAATAAAGAGCTGAATTC 780

***rhox2G*** TACTTGTATTTGCAATAAAGAGCTGAATTC 780

***rhox2H*** TACTTGTATTTGCAATAAAGAGCTGAATTC 780

**Additional File 2A:**

ClustalW alignment of predicted *rhox2A-H* cDNAs. Variable nucleotides are highlighted in blue. The start and stop codons are highlighted in green and red respectively.

***rhox3***

***rhox3A*** ATGAAGCCAGAACGTTCCATCTCTAACTGGATACATAGTAATGTGGAACGGGCTGGAAGAAATCTCTTCCAGGTC 75

***rhox3B*** ATGAAGCCAGAACGTTCCATCTCTAACTAGATACATAGTAATGTGGAACGGGCTGGAAGAAATCTCTTCCAGGTC 75

***rhox3C*** ATGAAGCCAGAACGTTCCATCTCTAACTGGATACATAGTAATGTGGAACGGGCTGGAAGAAATCTCTTCCAGGTC 75

***rhox3E*** ATGAAGCCAGAACGTTCCATCTCTAACTGGATACATAGTAATGTGGAACGGGCTGGAAGAAATCTCTTCCAGGTC 75

***rhox3E*** ATGAAGCCAGAACGTTCCATCTCTAACTGGATACATAGTAATGTGGAACGGGCTGGAAGAAATCTCTTCCAGGTC 75

***rhox3G*** ATGAAGCCAGAACGTTCCATGTCTAACTGGATACATAGTAATGTGGAACCGGCTGGAAGAAATCTCTTCCAGGTC 75

***rhox3F*** ATGAAGCCAGAACGTTCCATCTCTAACTAGATACATAGTAATGTGGAACCGGCTGGAAGAAATCTCTTCCAGGTC 75

***rhox3H*** ATGAAGCCAGAACGTTCCATCTCTAACTGGATACATAGTAATGTGGAACCGGCTGGAAGAAATCTCTTCCAGGTC 75

***rhox3A*** AACGGTCACCGCTCTGCTCTATTACCGGAACTACCTCAGGATTACCACAGAGCTTCAAGGTCAGTCAACGGATGT 150

***rhox3B*** AACGGTCACCGCTCTGCTCTATTACCGGAACTACCTCAGGATTACCACAGAGCTTCAAGGTCAGTCAACGGATGT 150

***rhox3C*** AACGGTCACCGCTCTGCTCTATTACCGGAACTACCTCAGGATTACCACAGAGCTTCAAGATCAGTCTACGGATGT 150

***rhox3D*** AACGGTCACCGCTCTGCTCTATTACCGGAACTACCTCAGGATTACCACAGAGCTTCAAGGTCAGTCAACGGATGT 150

***rhox3E*** AACGGTCACCGCTCTGCTCTATTACCGGAACTACCTCAGGATTACCACAGAGCTTCAAGGTCAGTCAACGGATGT 150

***rhox3F*** AACGGTCACCGCTCTGCTCTATTACCGGAACTACCTCAGGATTACCACAGAGCTTCAAGGTCAGTCAACGGATGT 150

***rhox3G*** AATGGTCACCGCTCTGCTCTATTACTGGAACTACCTCAGTATTACCACAGAGCTTCAAGGTCAGTCAACGGATGT 150

***rhox3H*** AACGGTCACCGCTCTGCTCTATTACCGGAACTACCTCAGGATTACCACAGAGCTTCAAGGTCAGTCAACGGATGT 150

***rhox3A*** GAGACTAAAATGGACAGCACCCAAGGTACCAAGGTTTTGCCGGCTGAAGAGGGAAAAAATGAAGAAGATGGAGGA 225

***rhox3B*** GAGACTAAAATGGACAGCACCCAAGGTACCAAGGTTTTGCCGGCTGAAGAGGGAAAAAATGAAGAAGATGGAGGA 225

***rhox3C*** GAGACTAAAATGGACAGCACCCAAGGTACCAAGGTTTTGCCGGCTGAAGAGGCAAGAAATGAGGAAGATGGAGGA 225

***rhox3D*** GAGACTAAAATGGACAGCACCCAAGGTACCAAGGTTTTGCCGGCTGAAGAGGGAAAAAATGAAGAAGATGGAGGA 225

***rhox3E*** GAGACTAAAATGGACAGCACCCAAGGTACCAAGGTTTTGCCGGCTGAAGAGGGAAAAAATGAAGAAGATGGAGGA 225

***rhox3F*** GAGACTAAAATGGACAACACCCAAGGTACCAAGGTTTTGCCGGCTGAAGAGGCAAGAAATGAGGAAGATGGAGGA 225

***rhox3G*** GAGACTAAAATGGACAGCACCCAAGGTACCAAGGTTTTGCCGGCTGAAGAGGGAAGAAATGAGGAAGATGGAGGA 225

***rhox3H*** GAGACTAAAATGGACAGCACCCAAGGTACCAAGGTTTTGCCGGCTGAAGAGTCAAGAAATGAGGAAGATGGAGGA 225

***rhox3A*** CAGGTGGAGTCGGCATTGGGAGCCACAGCCGCAAGGGGTAGAGGAAAAGAAGCATTAAATGGAGAGAGTCCCGCC 300

***rhox3B*** CAGGTGGAGTCGGCATTGGGAGCCACAGCCACAAGGGGTAGAGGAAAAGAAGCATTAAATGGAGAGAGTCCCGCC 300

***rhox3C*** CAGGTGGAGTCGGCATTGGGAGCCACAGCCGCAAGGGGTAGAGGAAAAGAAGCATTAAATGGAGAGAGTCCCGCC 300

***rhox3D*** CAGGTGGAGTCGGCATTGGGAGCCACAGCCGCAAGGGGTAGAGGAAAAGAAGCATTAAATGGAGAGAGTCCCGCC 300

***rhox3E*** CAGGTGGAGTCGGCATTGGGAGCCACAGCCGCAAGGGGTAGAGGAAAAGAAGCATTAAATGGAGAGAGTCCCGCC 300

***rhox3F*** CAGGTCGAGTCGGCATTGGGAGCCACAGCCGCAAGGGGTAGAGGAAAAGAAGCATTAAATGGAGAGAGTCCCGCC 300

***rhox3G*** CAGGTGGAGTCGGCATTGGGAGCCACAGCCGCAAGGGGTAGAGGAAAAGAAGCATTAAATGGAGAGAGTCCCGCC 300

***rhox3H*** CAGGTGGAGTCGGCATTGGGAGCCACAGCCGCAAGGGGTAGAGGAAAAGAAGCATTAAATGGAGAGAGTCCCGCC 300

***rhox3A*** GCTGCTGGCACTGCAGGCCTTGTAGAGGAAGACAGGAACAAGGAAGATGGTGGCACCAAGGGAGGTGAGAAGAAT 375

***rhox3B*** GCTGCTGGCACTGCAGGCCTTGTAGAGGAAGACAGGAACAAGGAAGATGGTGGCACCAAGGGAGGTGAGAAGAAT 375

***rhox3C*** GCTGCTGGCACTGCAGGCCTTGTAGAGGAAGACAGGAACAAGGAAGATGGTGGCACCAAGGGAGGTGAGAAGAAT 375

***rhox3D*** GCTGCTGGCACTGCAGGCCTTGTAGAGGAAGACAGGAACAAGGAAGATGGTGGCACCAAGGGAGGTGAGAAGAAT 375

***rhox3E*** GCTGCTGGCACTGCAGGCCTTGTAGAGGAAGACAGGAACAAGGAAGATGGTGGCACCAAGGGAGGTGAGAAGAAT 375

***rhox3F*** GCTGCTGGCACTGCAGGCCTTGTAGAGGAAGACAGGAACAAGGAAGATGGTGGCACCAAGGGAGGTGAGAAGAAT 375

***rhox3G*** GCTGCTGGCACTGCAGGCCTTGTAGAGGAAGACAGGAACAAGGAAGATGGTGGCACCAAGGGAGGTGAGAAGAAT 375

***rhox3H*** GCCGCTGGCACTGCAGGCCTTGTAGAGGAAGATAGGAACAAGGAAGATGGTGGCACCAAGGGAGGTGAGAAGAAT 375

***rhox3A*** GAGCAGGAAGTGAGGGAGCAGATTCCTGAGCATGTTGAAGGAGAGAGTGACCAGGCTGAAGCGCCAAGGCAGGTG 450

***rhox3B*** GAGCAGGAAGTGAGGGAGCAGATTCCTGAGCATGTTGAAGGAGAGAGTGACCAGGCTGAAGCGCCAAGGCAGGTA 450

***rhox3C*** GAGCAGGAAGTGAGGGAGCAGATTCCTGAGCATGTTGAAGGAGAGAGTGACCAGGCTGAAGCACCAAGGCAGGTG 450

***rhox3D*** GAGCAGGAAGTGAGGGAGCAGATTCCTGAGCATGTTGAAGGAGAGAGTGACCAGGCTGAAGCGCCAAGGCAGGTG 450

***rhox3E*** GAGCAGGAAGTGAGGGAGCAGATTCCTGAGCATGTTGAAGGAGAGAGTGACCAGGCTGAAGCGCCAAGGCAGGTG 450

***rhox3F*** GAGCAGGAAGTGAGGGAGCAGATTCCTGAGCATGTTGAAGGAGAGAGTGACCAGGCTGAAGCGCTAAGGCAGGTG 450

***rhox3G*** GAGCAGGAAGTGAGGGAGCAGATTCCTGAGCATGTTGAAGGAGAGAGTGACCAGGCTGAAGCGCCAAGGCAGGTG 450

***rhox3H*** GAGCAGGAAGTGAGGGAGCAGATTCCTGAGCATGTTGAAGGAGAGAGTGACCAGGCTGAAGCGCCAAGGCAGGTA 450

***rhox3A*** CCACGACGTCGATTGCACCATAGATTCACCCAGTGGCAGCTGGACGAACTGGAGAGAATTTTCCGGATGAATTAT 525

***rhox3B*** CCACGACGTCGATTGCACCATAGATTCACCCAGTGGCAGCTGGACGAACTGGAGAGAATTTTCCGGATGAATTAT 525

***rhox3C*** CCACGACGTCGATTGCACCATAGATTCACCCAGTGGCAGCTGGACGAACTGGAGAGAATTTTCCGGATGAATTAT 525

***rhox3D*** CCACGACGTCGATTGCACCATAGATTCACCCAGTGGCAGCTGGACGAACTGGAGAGAATTTTCCGGATGAATTAA 525

***rhox3E*** CCACGACGTCGATTGCACCATAGATTCACCCAGTGGCAGCTGGACGAACTGGAGAGAATTTTCCGGATGAATTAT 525

***rhox3F*** CCACGACGTCGATTGCACCATAGATTCACCCAGTGGCAGCTGGACGAACTGGAGAGAATTTTCCGGATGAATTAT 525

***rhox3G*** CCACGACGTCGATTGCACCATAGATTCACCCAGTGGCAGCTGGACGAACTGGAGAGAATTTTCCGGATGAATTAT 525

***rhox3H*** CCACGACGTCGATTGCACCATAGATTCACCCAGTGGCAGCTGGACGAACTGGAGAGAATTTTCCGGATGAATTAT 525

***rhox3A*** TTTCTCAGTCTAGAAGCAAGAAAACAACTGGCCCGATGGATGGGTGTGAATGAAGCCATAGTGAAGAGATGGTTT 600

***rhox3B*** TTTCTCAGTCTAGAAGCAAGAAAACAACTGGCCCGATGGATGGGTGTGAATGAAGCCATAGTGAAGAGATGGTTT 600

***rhox3C*** TTTCTCAGTCTAGAAGCAAGAAAACAACTGGCCCGATGGATGGGTGTGAATGAAGCCATAGTGAAGAGATGGTTT 600

***rhox3D*** TTTCTCAGTCTAGAAGCAAGTAAACAACTGGCCCGATGGATGGGTGTGAATGAAGCCATAGTGAAGAGATGGTTT 600

***rhox3E*** TTTCTCAGTCTAGAAGCAAGAAAACAACTGGCCCGATGGATGGGTGTGAATGAAGCCATAGTGAAGAGATGGTTT 600

***rhox3F*** TTTCTCAGTCTAGAAGCAAGAAAACAACTGGCCCGATGGATGGGTGTGAATGAAGCCATAGTGAAGAGATGGTTT 600

***rhox3G*** TTTCTCAGTCTAGAAGCAAGAAAACAACTGGCCCGATGGATGGGTGTGAATGAAGCCATAGTGAAGAGATGGTTT 600

***rhox3H*** TTTCTCAGTCTAGAAGCAAGAAAACAACTGGCCCGATGGATGGGTGTGAATGAAGCCATAGTGAAGAGATGGTTT 600

***rhox3A*** CAGAAGAGGAGAGAACAATACAGGTGGTATAAGAGGCTATAAGGTCTCAGAAGTTCTCCTCCTGCTTCTCAGAAC 675

***rhox3B*** CAGAAGAGGAGAGAACAATACAGGTGGTATAAGAGGCTATAAGGTCTCAGAAGTTCTCCTCCTGCTTCTCAGAAC 675

***rhox3C*** CAGAAGAGGAGAGAACAATACAGGTGGTATAAGAGGCTATAAGGTCTCAGAAGTTCTCCTCCTGCTTCTCAGAAC 675

***rhox3D*** CAGAAGAGGAGAGAACAATACAGGTGGTATAAGAGGCTATAAGGTCTCAGAAGTTCTCCTCCTGCTTCTCAGAAC 675

***rhox3E*** CAGAAGAGGAGAGAACAATACAGGTGGTATAAGAGGCTATAAGGTCTCAGAAGTTCTCCTCCTGCTTCTCAGAAC 675

***rhox3F*** CAGAAGAGGAGAGAAAAATACAGGTGGTATAAGAGGCTATAAGGTCTCAGAAGTTCTCCTCCTGCTTCTCAGAAC 675

***rhox3G*** CAGAAGAGGAGAGAACAATACAGGTGGTATAAGAGGCTATAAGGTCTCAGAGGTTCTCCTCCTGCTTCTCAGAAC 675

***rhox3H*** CAGAAGAGGAGAGAACAATACAGGTGGTATAAGAGGCTATAAGGTCTCAGAAGTTCTCCTCCTGCTTCTCAGAAC 675

***rhox3A*** ATCTTTCCTGAAGACTGTGGAGGAACCCTGCAGTGCCACTATCGCCAAGGCAACATAGAGAGAGGAATTGCTTTC 750

***rhox3B*** ATCTTTCCTGAAGACTGTGGAGGAACCCTGCAGTGCCACTATCGCCAAGGCAACATAGAGAGAGGAATTGTTTTC 750

***rhox3C*** ATCTTTCCTGAAGACTGTGGAGGAACCCTGCAGTGCCACTATCGCCAAGGCAACATAGAGAGAGGAATTTTTTTC 750

***rhox3D*** ATCTTTCCTGAAGACTGTGGAGGAACCCTGCAGTGCCACTATCGCCAAGGCAACATAGAGAGAGGAATTGTTTTC 750

***rhox3E*** ATCTTTCCTGAAGACTGTGGAGGAACCCTGCAGTGCCACTATCGCCAAGGCAACATAGAGAGAGGAATTGCTTTC 750

***rhox3F*** ATCTTTCATGAAGACTGTGGAGGAACCCTGCAGTGCAAATATTACCAAGGCAACAGAGAGAAATGAATTGCTTTC 750

***rhox3G*** ATCTTTCATGAAGACTGTGGAGGAACCCTGCAGTGCAAATATCACCAAGGCAACAGAGAGAAATGAATTGCTTTC 750

***rhox3H*** ATCTTTCCTGAAGACTGTGGAAGAACCCTGAAGTGCCACTATCGCCTAGGCAACATAGAGAGAGGAATTGTTTTC 750

***rhox3A*** GTCTCCTAACGATATGTTATTAAACTCTTATATCTGAAGCGATTATATTTCAATAACAATATGAATTTTCAATAT 825

***rhox3B*** TTCTCCTAACGATATGTTATTAAACTCTTATATCTGAAGCGATTATATTTCAATAACAATATGAATTTTCAATAT 825

***rhox3C*** GTTTCCTAACGATATGTTATTAAACTCTTATATCTGAAGCGATTATATTTCAATAACAATATGAATTTTCAATAT 825

***rhox3D*** TTCTCCTAACGATATGTTATTAAACTCTTATATCTGAAGCGATTATATTTCAATAACAATATGAATTTTCAATAT 825

***rhox3E*** ATCTCCTAACGATATGTTATTAAACTCTTATATCTGAAGCGATTATATTTCAATAACAATATGAATTTTCAATAT 825

***rhox3F*** TTCTCCTAACAATATGTTATTAAACTCTTATATCTGAAGCGATTATATTTCAATAACAATATGAATTTTCAATAT 825

***rhox3G*** TTCTCCTAACAATATGTTATTAAACTCTTATATCTGAAGCGATTATATTTCAATAACAATATGAATTTTCAATAT 825

***rhox3H*** TTCTCCTAACGTTATGTTATTAAACTCTTATATCTGAAGCGATTATATTTCAATAACAATATGAATTTTCAATAT 825

**Additional File 2B:**

ClustalW alignment of predicted *rhox3A-H* cDNAs. Variable nucleotides are highlighted in blue. The start and stop codons are highlighted in green and red respectively.

***rhox4***

***rhox4A*** GAACTCCGACTCAGAATCTGCTGGGGAAAGCTGCAGGGAAGCACTCAGGACATGGAGCATCAAAACACCAACTAC 75

***rhox4B*** GAACTCCGACTCAGAATCTGCTGGGGAAAGCTGCAGGGAAGCACTCAGGACATGGAGCATCAAAACACCAACTAC 75

***rhox4C*** GAGCTCCGACTCAGAATCTGCTGGGGAAAGCTGCAGGGAAGCCCTCAGGACATGGAGCATCAAAACACCAACTAC 75

***rhox4D*** GAACTCCGACTCAGAATCTGCTGGGGAAAGCTGCAGGGAAGCACTCAGGACATGGAGCATCAAAACACCAACTAC 75

***rhox4E*** GAACTCCGACTCAGAATCTGCTGGGGAAAGCTGCAGGGAAGCACTCAGGACATGGAGCATCAAAACACCAACTAC 75

***rhox4G*** GATGTCCGACTCAGATTCTGCTGGGGAAAGCTGCAGGGAAGCACTCAGGACATGGAGCATCAAAACACCAACTAC 75

***rhox4H*** GAGCTCCGACTCAGAATCTGCTGGGGAAAGCTGCAGGGAAGCACTCAGGACATGGAGCATCAAAACACCAACTAC 75

***rhox4A*** CTACTTCATGAGGGACTTGGCAAGGACAAGGAAAATTTGAATGGTGGGAAGACACAGGCAGTCTTACCACTGGAT 150

***rhox4B*** CTACTTCATGAGGGACTTGGCAAGGACAAGGAAAAGTTGAATGGTGGGAAGACACAGACAGTCTTACCACTGGAT 150

***rhox4C*** CTACTTCATGAGGGACTTGGCAAGGACAAGGAAAAGTTGAATGGTGGGAAGACACAGGCAGTCTTACCACTGGAT 150

***rhox4D*** CTACTTCATGAGGGACTTGGCAAGGACAAGGAAAATTTGAATGGTGGGAAGACACAGACAGTCTTACCACTGGAT 150

***rhox4E*** CTACTTCATGAGGGACTTGGCAAGGACAAGGAAAATTTGAATGGTGGGAAGACACAGGCAGTCTTACCACTGGAT 150

***rhox4G*** CTACTTCATGAGGGACTTGTCAAGGACAAGGAAAAGTTGAATGGTAGGAAGACACAGACAGTCTTACCACTGGAT 150

***rhox4H*** CTACTTCATGAGGGACTTGGCAAGGACAAGGAAAAGTTGAATGGTGGGAAGACACAGGCAGTCTTACCACTGGAT 150

***rhox4A*** GGAGAGGGAAGAAATGAGGGAGAGAGTGTACTGGGCCAGTCCGGAGCCGCAGCAGTGGAAGGGGACAAAGCAGAA 225

***rhox4B*** GGAGAGGGAAGAAATGAGGGAGAGAGTGTACTGGGCCAGTCCGGAGCCGCAGCAGTGGAATGGGACAAAGCAGAA 225

***rhox4C*** GGAGAGGGAAGAAATGAGGGAGAGAGTGTACTGGGCCAGTCCGGAGCCGCAGCAGTGGAAGGGGACAAAGCAGAA 225

***rhox4D*** GGAGAGGGAAGAAATGAGGGAGAGAGTGTACTGGGCCAGTCCGGAGCCGCAGCAGTGGAATGGGACAAAGCAGAA 225

***rhox4E*** GGAGAGGGAAGAAATGAGGGAGAGAGTGTACTGGGCCAGTCCGGAGCCGCAGCAGTGGAATGGGACAAAGCAGAA 225

***rhox4G*** GGAGAGGGAAGAAATGAGGGAGAGAGTGGACTGGGCCAGTCCGGAGCCACAGCAGTGGAAGGGGACAAAGCAGAA 225

***rhox4H*** GGAGAGGGAAGAAATGAGGGAGAGAGTGGACTGGGCCAGTCCGGAGCCGCAGCAGTGGAAGGGGACAAAGCAGAA 225

***rhox4A*** GAATTAAGTGGAGAAGGTGGGCCTGCTGCTGGTGATGCAGACCTCATGGATAACAGCAACCAAGAGGACCAGGAC 300

***rhox4B*** GAATTAAGTGGAGAAGGTGGGCCTGCTGCTGGTGATGCAGACCTCATGGATAACAGCAACCAAGAGGACCAGGAC 300

***rhox4C*** GAATTAAGTGGAGAAGGTGGGCCTGCTGCTGGTGATGCAGACCTCATGGATAACAGCAACCAAGAGGACCAGGAC 300

***rhox4D*** GAATTAAGTGGAGAAGGTGGGCCTGCTGCTGGTGATGCAGACCTCATGGATAACAGCAACCAAGAGGACCAGGAC 300

***rhox4E*** GAATTAAGTGGAGAAGGTGGGCCTGCTGCTGGTGATGCAGACCTCATGGATAACAGCAACCAAGAGGACCAGGAC 300

***rhox4G*** GAATTAAGTGGAGAAGGTGGGCCTGCTGCTGGTGATGCAGACCTCATGGATAACAGCAACCAAGAGGACCAGGAC 300

***rhox4H*** GAATTAAGTGGAGAAGGTGGGCCTGCTGCTGGTGATGCAGACCTCATGGATAACAGCAACCAAGAGGACCAGGAC 300

***rhox4A*** ACCAGTGGCAGTGCCCAGGAGGAGGAGAAGCTGCCAGAGGAGCCAGTTCTCAAGGATGCTGTGGTCATAGACAAA 375

***rhox4B*** ACCAGTGGCAGTGCCCAGGAGGAGGAGAAGCTGCCAGAGGAGCCAGTTCTCAGGGATGCTGTGGTCATAGACAAA 375

***rhox4C*** ACCAGTGGCAGTGCCCAGGAGGAGGAGAAGCTGCCAGAGGAGCCAGTTCTCAAGGATGCTGTGGTCATAGACAAA 375

***rhox4D*** ACCAGTGGCAGTGCCCAGGAGGAGGAGAAGCTGCCAGAGGAGCCAGTTCTCAGGGATGCTGTGGTCATAGACAAA 375

***rhox4E*** ACCAGTGGCAGTGCCCAGGAGGAGGAGAAGCTGCCAGAGGAGCCAGTTCTCAGGGATGCTGTGGTCATAGACAAA 375

***rhox4G*** ACCAGTGGCAGTGCCCAGGAGGAGGAGAAGCTGCCAGAGGAGCCAGTTCTCAAGGATGCTGTGGTCATAGACAAA 375

***rhox4H*** ACCAGTGGCAGTGCCCAGGAGGAGGAGAAGCTGCCAGAGGAGCCAGTTCTCAGGGATGCTGTGGTCATAGACAAA 375

***rhox4A*** GTGCAGCCTATTCCAGTGCTGGTATCTGGTGTGCGGCCTAAGTCAGTGTGGGTACAGCAGCGTAGCTTACACTAC 450

***rhox4B*** GTGCAGCCTATTCCAGTGCTGGTATCTGGTGTGCGGCCTAAGTCAGTGTGGGTACAGCAGCGTAGCTTACACTAC 450

***rhox4C*** GTGCAGCCTATTCCAGTGCTGGTATCTGGTGTGCGGCCTAAGTCAGTGTGGGTACAGCAGCGTAGCTTACACTAC 450

***rhox4D*** GTGCAGCCTATTCCAGTGCTGGTATCTGGTGTGCGGCCTAAGTCAGTGTGGGTACAGCAGCGTAGCTTACACTAC 450

***rhox4E*** GTGCAGCCTATTCCAGTGCTGGTATCTGGTGTGCGGCCTAAGTCAGTGTGGGTACAGCAGCGTAGCTTACACTAC 450

***rhox4G*** GTGCAGCCTATTCCAGTGCTGGTATCTGGTGTGCGGCCTAAGTCAGTGTGGGTACAGCAGCGTAGCTTACACTAC 450

***rhox4H*** GTGCAGCCTATTCCAGTGCTGGTATCTGGTGTGCGGCCTAAGTCAGTGTGGGTACAGCAGCGTAGCTTACACTAC 450

***rhox4A*** AATTTCCAATGGTGGCAGCTGCAGGAGCTGGAGCGCATTTTCCAGCAGAATCACTTCATCCGTGCAGAGGAAAGA 525

***rhox4B*** AATTTCCAATGGTGGCAGCTGCAGGAGCTGGAGCGCATTTTCCAGCAGAATCACTTCATCCGTGCAGAGGAAAGA 525

***rhox4C*** AATTTCCAATGGTGGCAGCTGCAGGAGCTGGAGCGCATTTTCCAGCAGAATCACTTCATCCGTGCAGAGGAAAGA 525

***rhox4D*** AATTTCCAATGGTGGCAGCTGCAGGAGCTGGAGCGCATTTTCCAGCAGAATCACTTCATCCGTGCAGAGGAAAGA 525

***rhox4E*** AATTTCCAATGGTGGCAGCTGCAGGAGCTGGAGCGCATTTTCCAGCAGAATCACTTCATCCGTGCAGAGGAAAGA 525

***rhox4G*** AATTTCCAATGGTGGCAGCTGCAGGAGCTGGAGCGCATTTTCCAGCAGAATCACTTCATCCGTGCAGAGGAAAGA 525

***rhox4H*** AATTTCCAATGGTGGCAGCTGCAGGAGCTGGAGCGCATTTTCCAGCAGAATCACTTCATCCGTGCAGAGGAAAGA 525

***rhox4A*** AGACATCTGGCAAGATGGATAGGTGTGAGTGAAGCCAGAGTTAAGAGATGGTTTAAGAAGAGGAGAGAGCACTTC 600

***rhox4B*** AGACATCTGGCAAGATGGATAGGTGTGAGTGAAGCCAGAGTTATGACATGGTTTAAGAAGAGGAGAGAGCACTTC 600

***rhox4C*** AGACATCTGGCAAGATGGATAGGTGTGAGTGAAACCAGAGTTAAGAGATGGTTTAAGAAGAGGAGAGAGCACTTC 600

***rhox4D*** AGACATCTGGCAAGATGGATAGGTGTGAGTGAAGCCAGAGTTATGAGATGGTTTAAGAAGAGGAGAGAGCACTTC 600

***rhox4E*** AGACATCTGGCAAGATGGATAGGTGTGAGTGAAACCAGAGTTAAGAGATGGTTTAAGAAGAGGAGAGAGCACTTC 600

***rhox4G*** AGACATCTGGCAAGATGGATAGGTGTGAGTGAAGCCAGAGTTATGACATGGTTTAAGAAGAGGAGAGAGCACTTC 600

***rhox4H*** AGACATCTGGCAAGATGGATAGGTGTGAGTGAAGCCAGAGTTATGACATGGTTTAAGAAGAGGAGAGAGCACTTC 600

***rhox4A*** AGAAGAGGACAAAGTCAGTTAGGAATGAATGATGACGCCTCTGTGGGGTCCCACTCTACCTTTCTCTGAAGATGG 675

***rhox4B*** AGAAGAGGACAAAGTCAGTTAGGAATGAATGATGATGCCCCTGTGGGGTCCCACTCTACCTTTCTCTGAAGATGG 675

***rhox4C*** AGAAGAGGACAAAGTCAGTTAGGAATGAATGACGATGCCCCTGTGGGGTCCCACTCTACCTTTCTCTGAAGATGG 675

***rhox4D*** AGAAGAGGACAAAGTCAGTTAGGAATGAATGATGATGCCCCTGTGGGGTCCCACTCTACCTTTCTCTGAAGATGG 675

***rhox4E*** AGAAGAGGACAAAGTCAGTTAGGAATGAATGATGATGCCCCTGTGGGGTCCCACTCTACCTTTCTCTGAAGATGG 675

***rhox4G*** AGAAGAGGACAAAGTCAGTTAGGAATGAATGATGATGCCCCTGTGGGGTCCCACTCTACCTTTCTCTGAAGATGG 675

***rhox4H*** AGAAGAGGACAAAGTCAGTTAGGAATGAATGATGATGCCCCTGTGGGGTCCCACTCTACCTTTCTCTGAAGATGG 675

***rhox4A*** CACAGGAGGCCTGGTGTACCACCCTTGACCAGGAGCCACGTGCAGACCCCTGCTGCCTTCCACCAGCATGTTATT 750

***rhox4B*** CACAGGAGGCCTGGTGTACCACCCTTGACCAGGAGCCACGTGCAGACCCCTGCTGCCTTCCACCAGCATGTTATT 750

***rhox4C*** CACAGGAGGCCTGGTGTACCACCCTTGACCAGGAGCCACGTGCAGACACCTGCTGTCTTCCACCAGCATGTTATT 750

***rhox4D*** CACAGGAGGCCTGGTGTACCACCCTTGACCAGGAGCCACATGCAGACACCTGCGGCCTTCCACCAGCATGTTATT 750

***rhox4E*** CACAGGAGGCCTGGTGTACCACCCTTGACCAGGAGCCACGTGCAGACCCCTGCTGCCTTCCACCAGCATGTTATT 750

***rhox4G*** CACAGGAGGCCTGGTGTACCACCCTTGACCAGGAGCCACGTGCAGACCCCTGCTGCCTTCCACCAGCATGTTATT 750

***rhox4H*** CACAGGAGGCCTGGTGTACCACCCTTGACCAGGAGCCACGTGCAGACCCCTGCTGCCTTCCACCAGCATGTTATT 750

***rhox4A*** GCATCTCTATTCCCTAAGCATTTGTATGTGAAATAAATAGATTCTCAGTTTCTTTG 806

***rhox4B*** GCATCTCTATTCCCTAAGCATTTGTATGTGAAATAAATAGATTCTCAGTTTCTTTG 806

***rhox4C*** GCAACTCTATTCCCTAAGCATTTGTATGTGAAATAAATAGATTCTCAGTTTCTTTG 806

***rhox4D*** GCATCTCTATTCCCTAAGCATTTGTATGTGAAATAAATAGATTCTCAGTTTCTTTG 806

***rhox4E*** GCATCTCTATTCCCTAAGCATTTGTATGTGAAATAAATAGATTCTCAGTTTCTTTG 806

***rhox4G*** GCATCTCTATCCCCTAAGCATTTGTATGTGAAATAAATAGATTCTCAGTTTCTTTG 806

***rhox4H*** GCATCTCTATCCCCTAAGCATTTGTATGTGAAATAAATAGATTCTCAGTTACTTTG 806

**Additional File 2C:**

ClustalW alignment of predicted *rhox4A-H* cDNAs. Variable nucleotides are highlighted in blue. The start and stop codons are highlighted in green and red respectively.
