# Supplementary material for: A murine specific expansion of the Rhox cluster involved in embryonic stem cell biology is under natural selection
Source: BMC Genomics. 2006 Aug 17;7:212. doi: 10.1186/1471-2164-7-212 (PMC1562416; doi:10.1186/1471-2164-7-212)
Supplement: Additional file 3 — Unique nucleotide profile of predicted Rhox3A-H transcripts and Clustal W alignment of predicted amino acid sequence from Rhox3A-H. [file 1471-2164-7-212-S3.pdf]

Rhox3

A

variant nucleotides

	-29	50	203	206	450	525	546	712	746	751
rhox3A	G	G	G	A	G	T	A	C	C	G
rhox3B	A	G	G	A	A	T	A	C	T	T
rhox3C	G	G	C	G	G	T	A	C	T	G
rhox3D	G	G	G	A	G	A	T	C	T	T
rhox3E	G	G	G	A	G	T	A	C	C	A
rhox3F	G	C	C	G	G	T	A	A	C	T
rhox3G	A	C	G	G	G	T	A	A	C	T
rhox3H	G	C	C	G	A	T	A	C	T	T

B

```
RHOX3A MKPERSISNWIHSNVERAGRNLFQVNGHRSALLPELPQDYHRASRSVNGCETKMDSTQGT 60
RHOX3B MKPERSISN--------------------------------------------------- 9
RHOX3C MKPERSISNWIHSNVERAGRNLFQVNGHRSALLPELPQDYHRASRSVYGCETKMDSTQGT 60
RHOX3D MKPERSISNWIHSNVERAGRNLFQVNGHRSALLPELPQDYHRASRSVNGCETKMDSTQGT 60
RHOX3E MKPERSISNWIHSNVERAGRNLFQVNGHRSALLPELPQDYHRASRSVNGCETKMDSTQGT 60
RHOX3F MKPERSMSNWIHSNVEPAGRNLFQVNGHRSALLPELPQDYHRASRSVNGCETKMDNTQGT 60
RHOX3G MKPERSISN--------------------------------------------------- 9
RHOX3H MKPERSISNWIHSNVEPAGRNLFQVNGHRSALLPELPQDYHRASRSVNGCETKMDSTQGT 60

RHOX3A KVLPAEEGKNEEDGGQVESALGATAARGRGKEALNGESPAAAGTAGLVEEDRNKEDGGTK 120
RHOX3B ------------------------------------------------------------ -
RHOX3C KVLPAEEARNEEDGGQVESALGATAARGRGKEALNGESPAAAGTAGLVEEDRNKEDGGTK 120
RHOX3D KVLPAEEGKNEEDGGQVESALGATAARGRGKEALNGESPAAAGTAGLVEEDRNKEDGGTK 120
RHOX3E KVLPAEEGKNEEDGGQVESALGATAARGRGKEALNGESPAAAGTAGLVEEDRNKEDGGTK 120
RHOX3F KVLPAEEARNEEDGGQVESALGATAARGRGKEALNGESPAAAGTAGLVEEDRNKEDGGTK 120
RHOX3G ------------------------------------------------------------ -
RHOX3H KVLPAEESRNEEDGGQVESALGATAARGRGKEALNGESPAAAGTAGLVEEDRNKEDGGTK 120
                                                  +  +   +   +
RHOX3A GGEKNEQEVREQIPEHVEGESDQAEAPRQVPRRRLHHRFTQWQLDELERIFRMNYFLSLE 180
RHOX3B ------------------------------------------------------------ -
RHOX3C GGEKNEQEVREQIPEHVEGESDQAEAPRQVPRRRLHHRFTQWQLDELERIFRMNYFLSLE 180
RHOX3D GGEKNEQEVREQIPEHVEGESDQAEAPRQVPRRRLHHRFTQWQLDELERIFRMN------ 174
RHOX3E GGEKNEQEVREQIPEHVEGESDQAEAPRQVPRRRLHHRFTQWQLDELERIFRMNYFLSLE 180
RHOX3F GGEKNEQEVREQIPEHVEGESDQAEALRQVPRRRLHHRFTQWQLDELERIFRMNYFLSLE 180
RHOX3G ------------------------------------------------------------ -
RHOX3H GGEKNEQEVREQIPEHVEGESDQAEAPRQVPRRRLHHRFTQWQLDELERIFRMNYFLSLE 180

           ++ ++ +    + *++**  *
RHOX3A ARKQLARWMGVNEAIVKRWFQKRREQYRWYKRL                            213
RHOX3B ---------------------------------                            -
RHOX3C ARKQLARWMGVNEAIVKRWFQKRREQYRWYKRL                            213
RHOX3D ---------------------------------                            174
RHOX3E ARKQLARWMGVNEAIVKRWFQKRREQYRWYKRL                            213
RHOX3F ARKQLARWMGVNEAIVKRWFQKRREKYRWYKRL                            213
RHOX3G ---------------------------------                            -
RHOX3H ARKQLARWMGVNEAIVKRWFQKRREQYRWYKRL                            213
```

Additional File 3

A. Unique nucleotide profile of predicted *rhox3A-H* transcripts. The position of the nucleotides is given relative to the published start site. B. Clustal W alignment of predicted amino acid sequence from *rhox3A-H*. Residues different from the consensus are highlighted in blue (conservative amino acid change) or red (non-conservative amino acid change). The homeobox domain is underlined with hydrophobic residues (+) crucial for homeodomain packing and residues involved in DNA binding (*) annotated.
