# Supplementary material for: A murine specific expansion of the Rhox cluster involved in embryonic stem cell biology is under natural selection
Source: BMC Genomics. 2006 Aug 17;7:212. doi: 10.1186/1471-2164-7-212 (PMC1562416; doi:10.1186/1471-2164-7-212)
Supplement: Additional file 4 — Analysis of transfection efficiency of E14/T ES cells with eGFP control and Rhox2 anti-sense constructs. [file 1471-2164-7-212-S4.pdf]

A

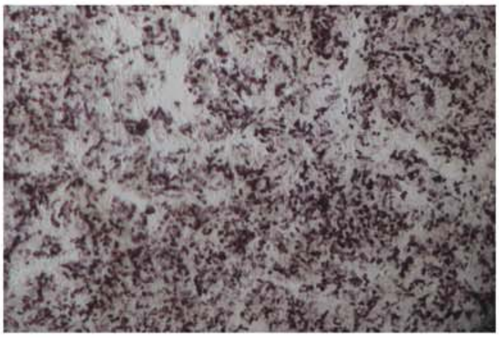

B

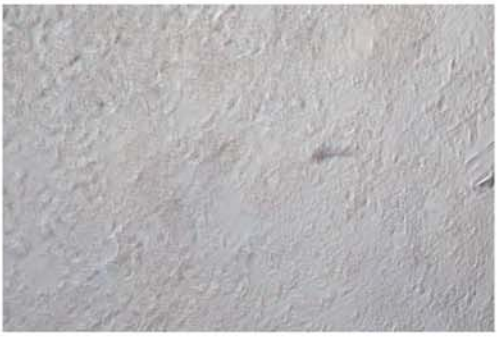

C

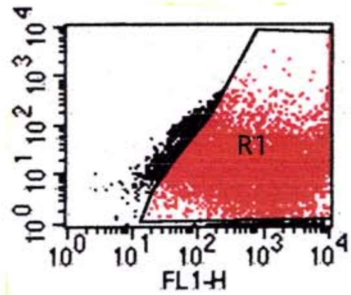

Additional file 4.

In Situ hybridisation of a digoxigenin labelled *Rhox2* labelled sense riboprobe to E14/T ES cells supertransfected with *Rhox2* anti-sense (A) or eGFP (B) expression constructs. (C) Flow cytometry of eGFP expressing cells demonstrate a 93% transfection efficiency.
